# Cumulative Neutral Loss Model for Fragment Deconvolution in Electrospray Ionization High-Resolution Mass Spectrometry Data

**DOI:** 10.1021/acs.analchem.3c00896

**Published:** 2023-08-07

**Authors:** Denice van Herwerden, Jake W. O’Brien, Sascha Lege, Bob W. J. Pirok, Kevin V. Thomas, Saer Samanipour

**Affiliations:** † Van ’t Hoff Institute for Molecular Sciences (HIMS), University of Amsterdam, Amsterdam 1012 WX, The Netherlands; ‡ Queensland Alliance for Environmental Health Sciences (QAEHS), The University of Queensland, Brisbane 4102, Australia; § Agilent Technologies Deutschland GmbH, Waldbronn 76337, Germany; ⊥ UvA Data Science Center, University of Amsterdam, Amsterdam 1012 WP, The Netherlands

## Abstract

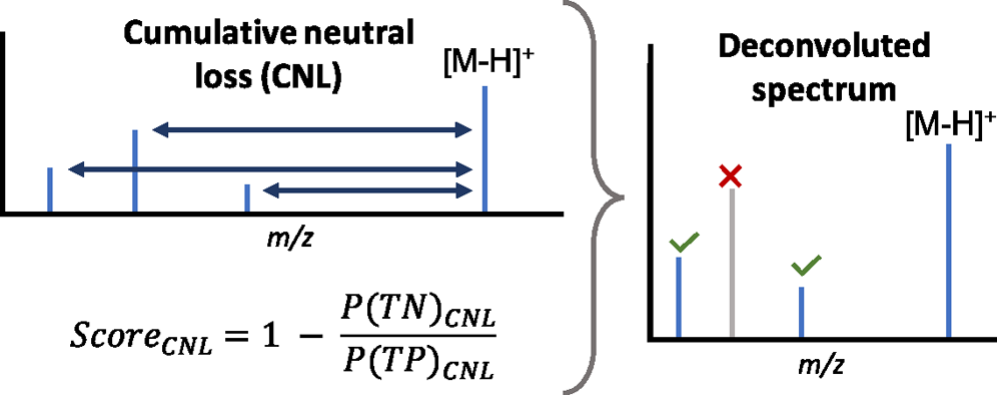

Clean high-resolution mass spectra (HRMS) are essential to a successful structural elucidation of an unknown feature during nontarget analysis (NTA) workflows. This is a crucial step, particularly for the spectra generated during data-independent acquisition or during direct infusion experiments. The most commonly available tools only take advantage of the time domain for spectral cleanup. Here, we present an algorithm that combines the time domain and mass domain information to perform spectral deconvolution. The algorithm employs a probability-based cumulative neutral loss (CNL) model for fragment deconvolution. The optimized model, with a mass tolerance of 0.005 Da and a score_CNL_ threshold of 0.00, was able to achieve a true positive rate (TPr) of 95.0%, a false discovery rate (FDr) of 20.6%, and a reduction rate of 35.4%. Additionally, the CNL model was extensively tested on real samples containing predominantly pesticides at different concentration levels and with matrix effects. Overall, the model was able to obtain a TPr above 88.8% with FD rates between 33 and 79% and reduction rates between 9 and 45%. Finally, the CNL model was compared with the retention time difference method and peak shape correlation analysis, showing that a combination of correlation analysis and the CNL model was the most effective for fragment deconvolution, obtaining a TPr of 84.7%, an FDr of 54.4%, and a reduction rate of 51.0%.

## Introduction

Nontargeted analysis (NTA) is a growing approach to uncover the known and unknown unknowns in complex samples, containing thousands of chemical constituents.^1−8^ Due to the complexity of the samples, coming from, for example, environmental or biological background, adequate data processing approaches are required for resolving the information belonging to unique chemical constituents.^1,4,7−21^ One of the most commonly used approaches to perform NTA is liquid chromatography coupled to high-resolution mass spectrometry (LC-HRMS). LC-HRMS, even though powerful in separating chemical constituents, is not able to fully resolve complex samples.^1,22^ This lack of full separation may result in overlapping signals from multiple chemicals (e.g., matrix signal), thus overlapping features in the MS1 signal. These overlapping features (i.e., MS1) are then further fragmented during MS2 signal generation, particularly in data-independent acquisition (DIA) experiments.^23^ These overlapping MS1 features result in a set of combined MS2 spectra, which may contain several false positive fragments.^4,17,19^ Therefore, steps to clean up (i.e., deconvolute) these spectra are warranted for highly confident and accurate identification of chemicals in complex samples.

Currently, the fragment deconvolution approaches (in DIA experiments) that are suitable for NTA focus heavily on the information presented in the time domain.^4,17,19,24^ One of these approaches is the apex retention time matching of the precursor features with the potential fragment signals.^4,11,13,16,17,25^ In addition to this, some algorithms also use peak shape assessment via correlation analysis to group fragments.^4,16−19,26,27^ More decomposition-based algorithms (e.g., MCR and/or PARAFAC) are available to perform signal deconvolution.^24,26,28^ These methods are generally less suitable for NTA due to the requirement of multiple samples, which often means that an analyte needs to be present in more than one sample and at different concentrations for the method to work. However, since it is unknown what is present in the samples to begin with, ensuring that the presence of this compound across multiple samples becomes impossible,^13,26,28,29^ even more so if different concentrations of the analyte are required to resolve the mass spectrum.^26^ Other reasons could be due to the requirement of retention time alignment, binning, prior knowledge on the number of components, or summation of MS1 and MS2 level information to obtain the fragment signals from the higher energy scan.^28^

Typically fragment deconvolution methods for NTA rarely utilize the mass domain information and are often highly field or compound class specific.^17,30,31^ Furthermore, in some cases (e.g., with data-dependent analysis), there could be a lack of time domain information, due to insufficient MS measuring points across the analyte peak, rendering the deconvolution of noise from the fragments impossible for these methods. Previous studies have shown that the mass domain can provide important information through, for example, neutral losses (NLs), which are defined as the mass differences between two ions, including precursor and fragment ions.^32,33^ Previously, NLs have been used for spectral annotation as well as molecular networking.^32,34,35^ These NLs are generated from the breakage of specific chemical bonds during ionization,^36^ hence their use in the reaction pathways for identification. However, if one fragment is missing from the fragment pattern, a different NL is obtained between the surrounding fragments. Hence, we propose the use of cumulative neutral losses (CNLs), which are defined by the difference between the precursor ion and each of the potential fragment ion masses.

In this paper, we present a probabilistic CNL model that only requires information from the mass domain for the deconvolution of fragment ions for small molecules (<1000 Da). The performance of the CNL model was evaluated for both fragments from spectral databases (i.e., MassBank EU, MassBank of North America (MoNA), and NIST20) and measurements containing predominantly pesticide standards. The latter were also evaluated at different sample concentrations and with varying levels of the added background matrix. Moreover, the model performance was compared with two frequently used methods, including peak apex retention time difference and peak shape correlation.

## Experimental Section

### Chemicals

Deionized water was produced onsite with a Milli-Q Integral 3 unit from MilliporeSigma (Germany). Acetonitrile (gradient grade for liquid chromatography, LiChrosolv) and ethanol (absolute for analysis, EMSURE) were purchased from MilliporeSigma (Germany). Formic acid (≥99%, HiPerSolv CHROMANORM for LC-MS) was obtained from VWR Chemicals (Germany). The LC/MS Pesticide Comprehensive Test Mix Kit (PN 5190-0551), consisting of eight individual submixtures of pesticides with a typical analyte concentration of 100 mg/L, was purchased from Agilent Technologies. Furthermore, mixtures of X-ray contrast media (syn. Radiopaque agents, mix 5), antibiotics (mix 6), and pharmaceuticals (mix 17) were obtained from Neochema (Germany) and the concentration of analytes in these individual mixtures was also 100 mg/L.

### Sample Preparation

A tea extract was prepared by sonicating two tea bags with a dry weight of 1.75 g each (black tea Klassik and rooibos tea Rooibos Vanille from Teekanne, Germany) in 100 mL of a water/ethanol solution (50%:50%, v/v) for 25 min. Afterward, the extract was filtered through a Captiva syringe filter (0.2 μm pore size, regenerated cellulose, Agilent Technologies). Blank solutions serving as diluent and negative controls were either a filtered water/ethanol solution (50%:50%, v/v, Blank A) or filtered tea extract that was further diluted 1:10 (Blank B) or 1:100 (Blank C) with Blank A. The three mixtures from Neochema were pooled together and diluted with Blank A to reach a final concentration of 1000 μg/L for each analyte. The eight submixtures of the comprehensive pesticide mix kit were kept initially separate and diluted with Blank A to final analyte concentrations of 10, 100, and 1000 μg/L. In addition, all eight pesticide submixtures were pooled together and diluted with Blank A, B, or C to final concentrations of 1, 2.5, 5, 10, 25, 50, 100, and 1000 μg/L.

### LC-ESI-Q-TOF Analysis

Chromatographic separation of analytes was performed using a 1290 Infinity II LC system (Agilent Technologies, Germany), consisting of a binary pump (G7120A), an autosampler (G7129B), and a column oven (G7116B). Samples were kept in the autosampler at room temperature (ca. 20 °C) and the injection volume was usually 1 μL. Analytes were separated on a Poroshell EC-C18 column (2.1 mm × 150 mm, 2.7 μm, Agilent Technologies) at a constant flow rate of 0.5 mL/min and a column temperature of 40 °C. The mobile phases were water + 0.1% formic acid (A) and acetonitrile + 0.1% formic acid (B). The following gradient program was used for the separation of analytes: at 0 min, 95% A; at 1 min, 95% A; at 21 min, 5% A; at 23 min, 5% A; at 23.1 min, 95% A; and at 28 min, 95% A. The HPLC system was connected to a G6546A quadrupole time-of-flight (Q-TOF) mass spectrometer (Agilent Technologies), equipped with an electrospray ionization (ESI) source using the Dual Spray Agilent Jet Stream technology. The Q-TOF was operated in the high-resolution mode for the low (*m*/*z* 1700) mass range. The acquisition rate was set to 6 Hz performing all ion full scan measurements at alternating collision energies of 0, 20, and 40 eV. Data were always recorded for a mass range of *m*/*z* 50–1200 in profile storage mode. Ionization was performed in the positive mode and the ESI source was operated under the following conditions: a drying gas temperature of 225 °C, a drying gas flow of 12 L/min, a sheath gas temperature of 350 °C, a sheath gas flow of 11 L/min, and a nebulizer pressure of 35 psi. The capillary and nozzle voltages were kept at 3500 and 500 V, respectively. A reference solution, containing purine and hexakis(1*H*,1*H*,3*H*-tetrafluoropropoxy)phosphazine (HP-0921), was continuously supplied to the second sprayer of the ESI source using an isocratic pump (G7110B, Agilent Technologies, Germany).

### Cumulative Neutral Loss Model

The CNL model was built based on the MassBank EU, MoNA, and NIST20 database entries that were obtained using electrospray ionization in positive mode with a mass resolution ≥5000, including any type of mass analyzer (i.e., Q-TOF and Orbitrap) and collision energy.^37−39^ Where multiple entries for a single chemical were found, the spectra were merged to ensure that the CNLs for each compound would have equal contribution to the model, using a 0.001 Da mass window. It should be noted that 0.001 Da was not assumed to be the inherent uncertainty of the data and was used as bin width for generation of average spectra. To obtain the CNLs for each compound, the fragment *m*/*z* values from the merged spectra were subtracted from the precursor ion mass. This resulted in reducing the 360,750 individual spectra to 24,487 merged CNL spectra for unique chemical constituents. The CNLs were used in place of the fragments to better capture the structural information implicitly present in the spectra. For example, while a high-frequency fragment may not contain much structural information, a highly frequent CNL provides information on the parts of the structure that are detached during fragmentation.^40^ For the CNL model building, a Bayesian (i.e., probabilistic) approach was employed to overcome the issues related to limited database and potential data leakage.^41^

**Figure 1:**
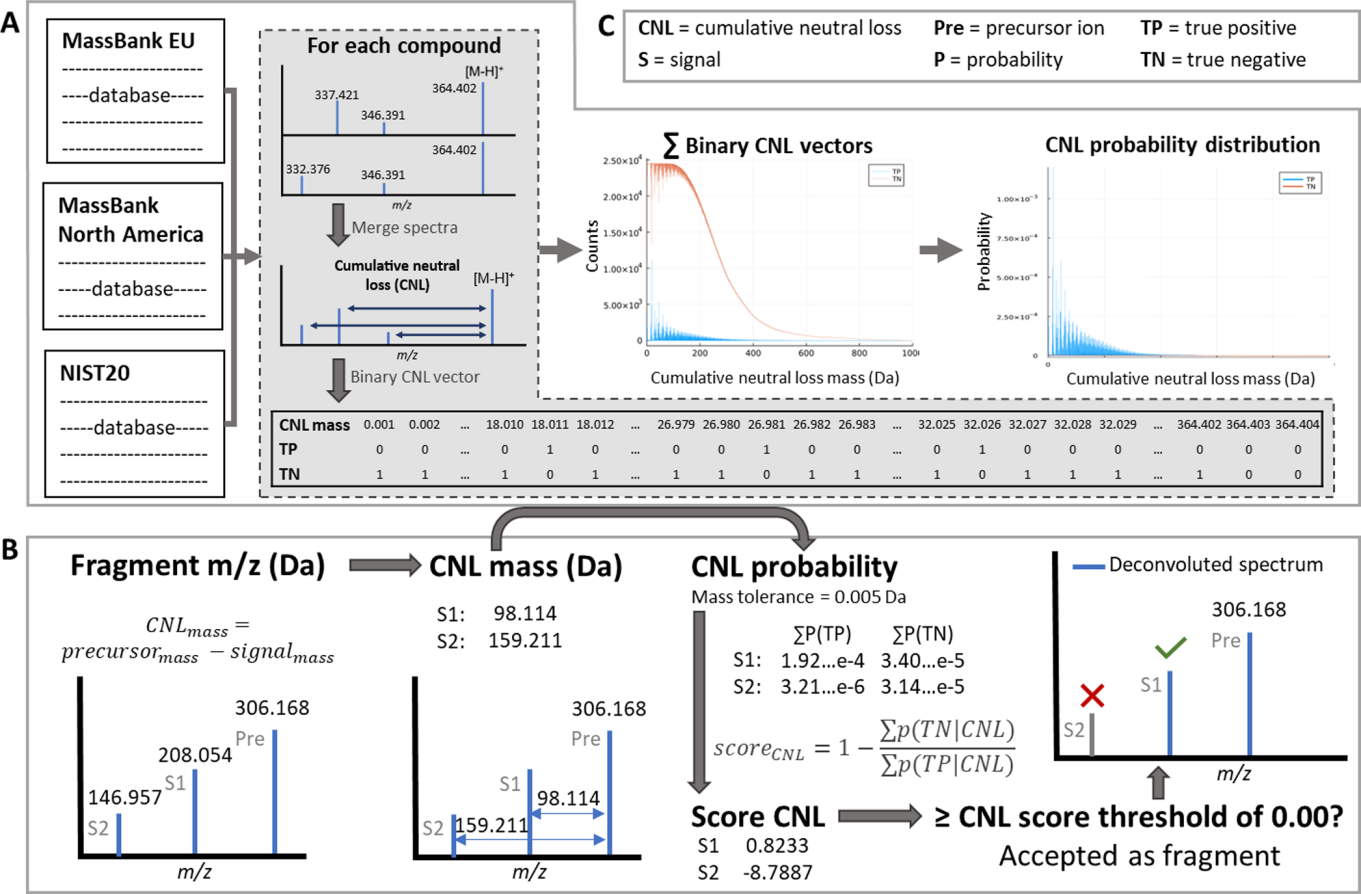
Workflow figure for construction of the CNL model (A) and an example calculation for using the CNL model with a mass tolerance of 0.005 Da and a score_CNL_ threshold of 0.00 (B). The abbreviations are listed in (C).

These CNL spectra were used to build the true positive (TP) and true negative (TN) probability distributions that were required for the Bayesian CNL model. To calculate these, the CNL spectra were converted to binary vectors for a CNL range of 0–1000 Da with 0.001 Da steps (i.e., 1,000,001 CNL values). The TP binary vector, for each chemical, contains ones for CNLs found in the spectra and zeros for the remaining bits, while the TN binary vector contains ones for the CNLs that were not found in a spectrum (Figure 1A). The CNL masses that were larger than the precursor ion were set to zero for the TN binary vector. The TP and TN CNL occurrence distributions were calculated by summing the binary vectors obtained for all 24,487 CNL spectra and adding 1 to each CNL bin to avoid obtaining probabilities equal to 0. Finally, the TP and TN probability distributions were obtained by dividing each CNL occurrence by the total number of TP and TN CNL occurrences, respectively. Overall, building the CNL model took about 1–2 h and depended on the number of spectra and unique chemicals in the databases

These calculated TP and TN CNL probabilities were used as the conditional probability (i.e., *P*(*B*|*A*)) in the Bayes theorem (eq 1). Additionally, to be able to calculate the TP or TN probability of a precursor ion having a specific CNL (i.e., posterior probability or *P*(*A*|*B*)), a flat prior (*P*(*A*)) is assumed, and since the marginal probability (*P*(*B*)) is a constant normalizing factor, the Bayes theorem can be reduced to eq 2, meaning that the TP and TN probabilities given a certain CNL are proportional to the probability of a CNL given that it is a TP or TN.
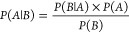
1
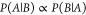
2

Through eq 2, the TP and TN probabilities for a CNL can be obtained, which were used to calculate score_CNL_ (eq 3). This score is used to evaluate whether a CNL belongs to a specific precursor ion mass. To calculate score_CNL_, the TP and TN sums of probability (i.e., ∑*P*(TP|CNL) and ∑*P*(TN|CNL), respectively) are obtained for specified CNL ± mass tolerance. For example, if the mass tolerance is set to 0.010 Da and a CNL mass of 18 has been found, then ∑*P*(TP) corresponds to the summed TP probabilities of the CNL masses of 17.99 to 18.01. After similarly calculating ∑*P*(TN), score_CNL_ can be used to assess if the CNL in question relates to a true fragment mass of the precursor ion mass or not, corresponding to above or below the score_CNL_ threshold, respectively. An example of calculating score_CNL_ is depicted in Figure 1B.
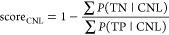
3

### CNL Model Performance

**Figure 2:**
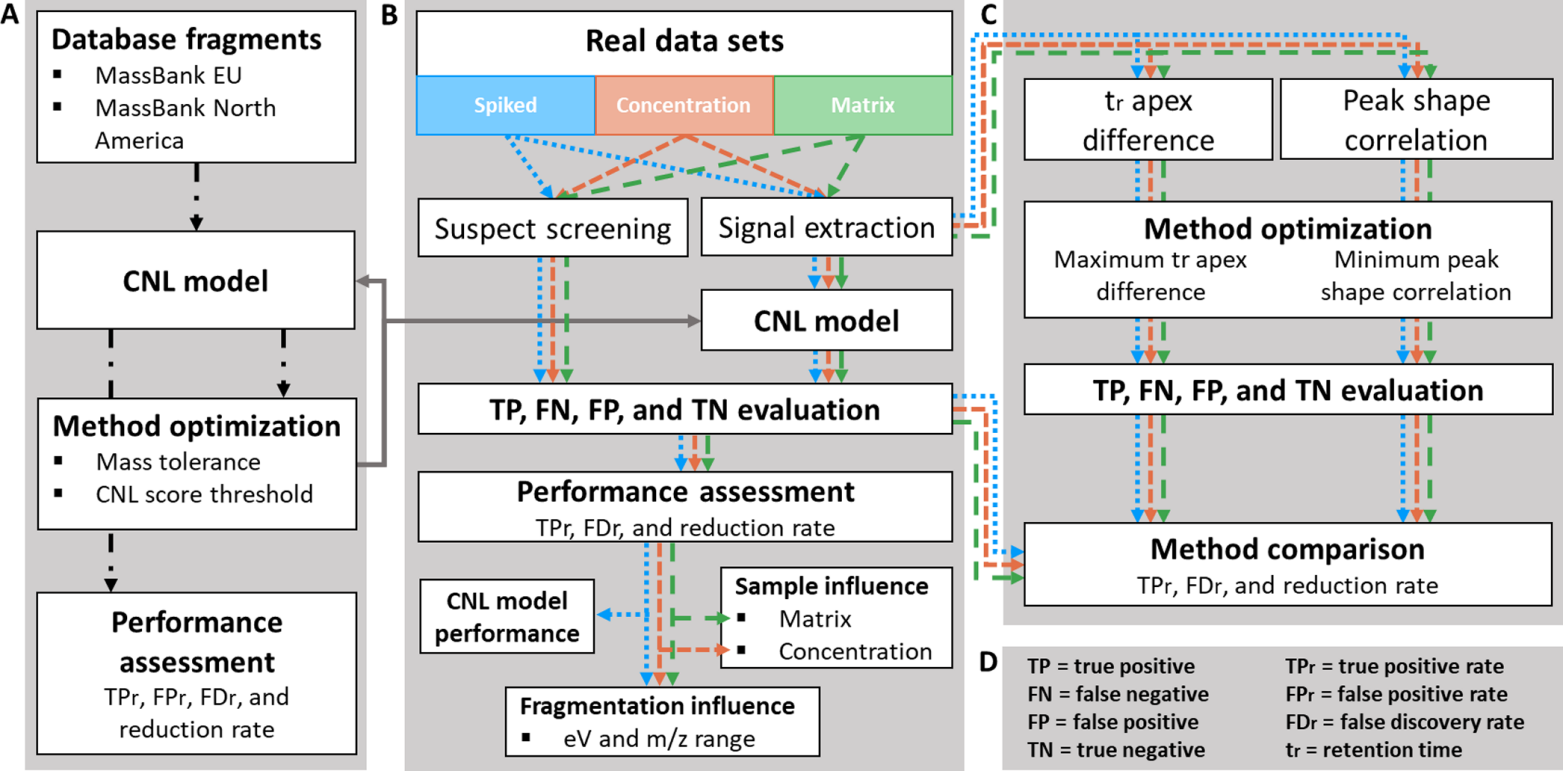
Workflow figure for the performance assessment of the CNL model with an overview of the data sets and what is used for which assessment. (A) Model optimization, (B) testing of the model with measured data, (C) comparison of the CNL model with the apex *t*_r_ difference and peak shape correlation method, and (D) the abbreviations are listed.

To assess the overall performance of the CNL model, multiple data sets have been used for different purposes. Figure 2 shows an overview of the assessment approaches and which data sets have been used. First, the model was trained and optimized using MassBank EU, MoNA, and NIST20 (Figure 2A). Second, three data sets (i.e., standard, matrix, and concentration) of real data were used to test the model and gain insights into the aspects influencing the performance (Figure 2B). The standard set was used to assess the general performance, the matrix set was used to assess the influence of matrix effects, and the concentration set was used to assess the influence of sample concentration, and for assessing the influence of the CNL mass range and collision energy, all of the data sets were used. Finally, all three data sets were also used for comparing the performance of the CNL model with existing techniques, namely, apex *t*_r_ difference and peak shape correlation analysis (Figure 2C).

#### CNL Model Performance Assessment for Database Fragments

For the model assessment, we focused on the raw data, TP and TN fragments, to avoid any biases introduced by the identification workflow. This process enabled us to thoroughly and objectively evaluate the performance of the developed algorithm. To carry out this evaluation, TP and TN fragment cases were obtained from databases. For this, the 360,750 measured spectra from MassBank EU, MoNA, and NIST20 were used.^37,38^ The TPs here were the CNLs generated from the true experimentally assessed fragments of a chemical. As for the TN CNLs, for each spectrum, 200 random masses (i.e., between 50 Da and precursor *m*/*z*) were generated and checked against all spectra of the compound in question to ensure that no *m*/*z* value of the random TN fragments was actually a true fragment of that compound. Then, the randomly generated *m*/*z* values were converted to TN CNL values. Overall, a total of 3,013,769 TP CNLs and 3,950,288 TN CNLs were obtained via the above-mentioned approach.

These cases were used to assess the influence of the CNL mass tolerance and score_CNL_ threshold on the model performance, using the TP, false positive (FP), and false discovery (FD) rates (eqs 4, 5, and 6, respectively). Both FP and FD rates were calculated, since they represent different information about the performance assessment and to be able to compare the results of the database fragment with the real data (i.e., the measured matrix containing predominantly pesticide standards). The FP rate represents the percentage of TN fragments that are wrongly accepted as true fragments and requires the number of TN fragments, which can be generated for the database spectra but are difficult to assess for the real data, whereas the FD rate represents the probability of an accepted fragment actually being a false positive case, which is calculated without using the number of TNs. These rates were calculated for CNL mass tolerances of 0.001, 0.005, 0.010, 0.020, 0.050, 0.100, and 0.200 Da and score_CNL_ thresholds ranging from −1 to 1 with steps of 0.01. Here, the TP and false negative (FN) cases correspond to TP CNLs that have a score_CNL_ above and below the score_CNL_ threshold, respectively. On the other hand, the FP and TN cases correspond to the TN CNLs that have a score_CNL_ above and below the score_CNL_ threshold, respectively. Finally, TP vs FP or FD rates were used in receiver operator curves (ROCs) to obtain the most optimal parameters, which are included in the Supporting Information.
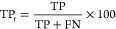
4
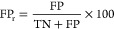
5
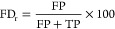
6

#### Data Processing for Real Samples

Both for the general performance evaluation of the CNL model and the comparison with conventional methods, potential fragment signals are needed to be extracted from all of the data files and processed using these fragment deconvolution methods. Therefore, in this section, the general data preprocessing method will be described for all performance evaluations using real samples. The raw data files were converted with MSConvertGUI (64-bit, ProteoWizard^42^) to the mzXML format, using a 100 counts per second absolute intensity threshold. MS1 feature lists were obtained for these files using the self-adjusting feature detection (SAFD) algorithm.^9^ The following settings were used: 10,000 maximum number of iterations, 20,000 resolution, minimum intensity of 500 counts, minimum mass domain window size of 0.02 Da, 0.75 minimum regression coefficient, a maximum signal increment of 5%, a signal-to-background ratio of 2, and an allowed peak width in the time domain between 3 and 200 scans.

For each MS1 feature list, the presence of the spiked reference compounds was checked. If the reference precursor ion mass with a mass tolerance of 0.010 Da was found in the MS1 feature list, then the corresponding MS2 signal masses were extracted. The set mass tolerance has been shown to be effective for such analysis when dealing with Q-TOF instruments with a nominal resolution of 30,000.^1^ To do so, first, the MS2 spectra within the start and end time of the MS1 feature were centroided with the centroiding algorithm from the SAFD package, using a minimum intensity of 250 counts and a resolution of 20,000.^9,43^ Second, the centroided MS2 spectra were used to obtain XICs for the present *m*/*z* values. The *m*/*z* values within a mass window of 0.020 Da (i.e., 0.010 mass tolerance) were grouped as a single XIC. Third, the MS2 XICs went through two quality control criteria, which meant that the signal-to-noise ratio needed to be higher than 2, and at least 3 consecutive scans were needed to have a higher intensity for both the forward and backward cumulative intensity means of XICs. The signals that met those criteria were considered for further evaluation. These filtered signals went through three processes in parallel to generate the data for the comparison of the CNL model to the conventional approaches. These processes were profile correlation analysis,^44^ apex retention time matching (*t*_r_), and CNL calculations.

On the other hand, suspect screening was performed to obtain the true fragments that were present in the measurements for the reference chemicals (Table S1), for which the Universal Library Search Algorithm (ULSA)^4^ was used. The suspect screening extracted the specified fragments in our suspect list based on checking their minimum intensity, retention time match, and the correlation coefficient between the XIC of the parent ion and the potential fragments. This enabled us to avoid the inclusion of interfering signals as well as false positive fragments. The settings for this algorithm were as follows: a mass tolerance of 0.010 Da, a *t*_r_ tolerance of 0.5 min, and a minimum MS1 intensity of 2000. Additionally, a suspect list of the reference compounds was required, containing a collection of fragments that were found in the MassBank EU, MoNA, and NIST20 spectra with a resolution of above 5000 for each of these compounds.

For the performance assessment of the CNL model and the conventional methods, TP and FD rates were calculated (eqs 4 and 6, respectively). Each of the three methods has a different threshold requirement for accepting or rejecting an *m*/*z* value as a fragment ion of the precursor ion. For the CNL model, score_CNL_ needs to be above the score_CNL_ threshold, the correlation needs to be above the correlation threshold, and the peak apex time difference needs to be below the maximum *t*_r_ difference. To calculate the detection rates (eqs 4 and 6), the TP and FN cases are the true fragments (i.e., found in the suspect screening list) that are accepted and rejected by a fragment deconvolution method, respectively. As for FP cases, these were fragments that were not present in the suspect screening list and were accepted as fragments. Finally, the MS1 feature lists, the extracted MS2 signals for the reference compounds, the suspect screening results, and the suspect list can be found on Figshare.^45^

#### CNL Model Performance for Real Samples

Besides evaluating the general CNL model performance, multiple aspects that can influence these results are also evaluated and discussed. However, for the general CNL performance, the 1000 μg/L spiked Neochema and pesticide samples with no other effects were used. These samples were used to select the optimum mass tolerance and score_CNL_ threshold for the model. This was achieved by setting up ROC curves for mass tolerances of 0.001, 0.005, 0.010, 0.020, 0.100, and 0.200 Da with score_CNL_ thresholds in a range of −1 to 1 with steps of 0.01. With the optimized mass tolerance selected, the influence of the sample concentration and matrix were assessed through ROC curves with the same score_CNL_ threshold range, using the pesticide samples at different concentrations and with the added tea matrix. Additionally, the difference in model performance for the lower and higher collision energy scans and for different ranges of CNLs was investigated, again using the optimized mass tolerance and score_CNL_ and all three sample sets. For the CNL range influence, the TP and FD rates were calculated for the cases with CNL mass ranges of 0–100, 100–200, 200–300, and 300–400 *m*/*z*.

#### Comparison with Conventional Methods

To compare the CNL model performance with currently available methods for fragment deconvolution, TP and FD rates were compared for the individual methods and combinations of these methods. However, first, the optimal correlation threshold and maximum apex *t*_r_ difference parameters were obtained, which were extracted by ROCs with a correlation threshold range of −1 to 1 with steps of 0.005 and a maximum *t*_r_ difference range of 0–0.6 with steps of 0.005. After the optimization of the conventional methods, the TP and FD rates were calculated for the combination of these methods to compare their performance and evaluate the optimal combination.

### Calculations and Code Availability

The calculations and model development were executed on a personal computer with 12 CPUs and 32 GB of RAM, using Windows 10. The CNL model was developed and evaluated with the Julia programming language (v1.6). The code for using the CNL model is available at https://bitbucket.org/Denice_van_Herwerden/cnlforfragments.jl/src/main/. This package also allows the user to reconstruct the CNL model as more spectra are added to the databases over time. Additionally, the MS_Import package was used for importing the mzXML files (https://bitbucket.org/SSamanipour/ms_import.jl/src/master/), the SAFD package for obtaining the MS1 feature lists (https://bitbucket.org/SSamanipour/safd.jl/src/master/), and the ULSA package was used for generating the suspect lists (https://bitbucket.org/SSamanipour/ulsa.jl/src/master/).

## Results and Discussion

### Exploring the CNL Model

**Figure 3:**
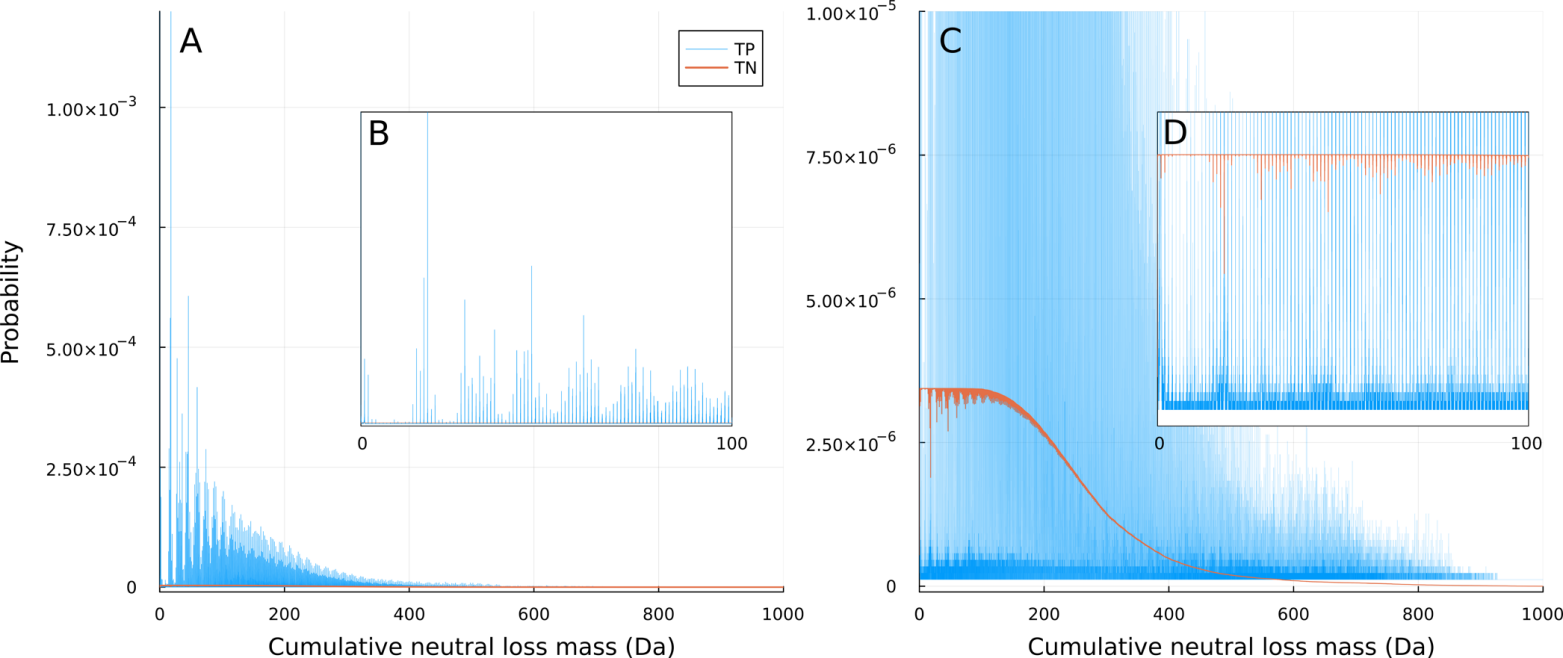
TP and TN CNL probability distributions that are implemented in the CNL model. (A) Full probability distribution, (B) a zoomed-in fraction of the TP probability distribution, and (C, D) the TN probability distribution zoomed-in on the probability range and CNL range, respectively.

When building the CNL model, the TP and TN counts for each CNL were calculated. At first sight, a general trend of a decrease in the total number of CNL counts after a CNL of 125 Da was found (Figure S1). This was expected, since the median of all precursor ions is ±300 Da and the higher CNLs are not possible (i.e., precursor ion mass < CNLs) for more and more compounds. Additionally, a distribution of the fragment *m*/*z* counts was also generated for comparison (Figure S2). Here, it can be seen that the fragment *m*/*z* counts are less clearly defined than the CNL distribution (Figure 3). Finally, the CNL counts were converted to probabilities, and still, the same trend was observed (Figure 3). However, the probabilities in the TN distribution are much smaller than the TP probability distribution due to the larger number of TN cases than TPs. Therefore, score_CNL_ is calculated that evaluates the relationship between *P*(TP) and *P*(TN).

To provide an idea of what these probabilities mean in terms of chemical information, an overview of a few CNL masses with high TP probability can be found in Table S2 with the potential CHNO compositions of these CNL masses. The most prominent CNL has been found at a mass of 18.01, which corresponds to a commonly known neutral loss of water or H_2_O. Other frequently occurring losses such as ammonia (NH_3_), methanol (CH_4_O), and, when looking at larger CNLs, C_2_H_4_O_2_ correspond to CNLs of 17.03, 32.03, and 60.02, respectively. These examples show that the CNL masses contain valuable information related to the neutral losses of the precursor ion, meaning that a fragment mass might not have the same *m*/*z* value for multiple precursor ions, while their CNL mass with that fragment is actually the same. Moreover, the CNL occurrence probabilities could potentially be used to assist fragmentation pattern prediction tools, by, for example, ranking the fragments based on their CNL occurrence probability.

### CNL Model Performance for Database Fragments

To assess the performance of the CNL model for database fragments, ROC curves were constructed for different mass tolerances (Figures S3 and S4). The ROC curves do not go up to 100% TP and FP rates because the lowest score_CNL_ threshold evaluated is −1, which does not correspond to the highest possible TPr. Overall, the 0.005 Da mass tolerance performed the best at a score_CNL_ threshold of 0.00, which had TP, FP, and FD rates of 95.0, 15.8, and 20.6%, respectively. Using the optimized parameters, the model was able to identify 95.0% of the correct cases and only 15.8% of the wrong cases as fragments. Moreover, of all CNLs that were identified as fragments, 20.6% were wrongly classified as fragments. A total of 4,722,913 cases were evaluated, of which 54.0% were true database fragments (i.e., almost a 1:1 ratio of TP and TN cases). Overall, 1,672,646 cases were removed by the CNL model, resulting in a reduction rate of 35.4% of the total number of potential fragments. Finally, using the selected mass tolerance of 0.005 Da and a score_CNL_ threshold of 0.00, it was shown that the CNL model was very well able to reduce the total number of potential fragments while retaining 95.0% of the true cases and eliminating a large portion (i.e., 84.2%) of the false fragments.

### CNL Model Performance for Real Samples

To evaluate the performance of the CNL model for real samples, the detection rates were obtained for the same ROC parameters as the database data, using the measurements spiked with standard mixtures. Instead of evaluating both FPr and FDr, only FDr is used when dealing with real data, since the number of TN detected fragments is not easily defined and is highly dependent on the sample (e.g., matrix) and noise in the spectrum. Using the optimized parameters from the database performance assessment (i.e., 0.005 Da mass tolerance and a score_CNL_ threshold of 0.00), a TPr of 98.6% and an FDr of 44.7% were found. For the other mass tolerances, similar FD rates were found except for 0.001 Da mass tolerance. Figure S5 shows that if the mass window is set to 0.001 Da, FDr increases to 49.1% at a score_CNL_ threshold of 0.00. These results showed that based on the current data set, a mass tolerance of 0.001 Da is at the boundary of the applicability. In general, a higher FDr was found for the real samples compared to the database fragments as well as a lower overall reduction rate of 8.2%. While the total number of evaluated cases, 5526, is significantly lower than the almost 5 million cases for the database fragments, a similar 1:1 ratio of correct and wrong fragment cases is observed.

Figure S6 shows the difference between the raw and deconvoluted spectrum for a single standard. From this, it can also be seen that the model does not distinguish between higher and lower intensity signals for accepting or rejecting these as CNLs. Additionally, TP, FN, FP, and TN fragment masses were investigated. For the TP (Figure S7) and FP (Figure S8) cases, it can be seen that the score_CNL_ for the *m*/*z* of these signals is above the score_CNL_ threshold. However, the FP fragment with an *m*/*z* of 160.974 Da was not recorded in the databases that were used for constructing the suspect list. This signal could be an unrecorded fragment *m*/*z* for this compound in the databases, noise, or a background signal with a high probability of being a fragment of the precursor in question. As for the TN (Figure S9) and FN (Figure S10) cases, score_CNL_ is below the score_CNL_ threshold.

#### Sample Influence

Additionally, the influence of the standard concentration and presence of a sample matrix on the CNL model performance was evaluated, using the same approach. For testing the matrix influence, the tea matrix was spiked into the samples at different dilution factors (i.e., no matrix, 100× diluted, and 10× diluted) with varying standard concentrations (i.e., 1, 2.5, 5, 10, 25, 50, 100, 1000 μg/L). Using the selected score and mass tolerance and combining the results with different standard concentrations, TPr values of 81.1, 82.5, and 82.1%, FDr values of 69.5, 75.4, and 75.4%, and reduction rates of 33.1, 31.7, and 32.9% were obtained for the data sets without the matrix, with a 100× diluted matrix, and with a 10× diluted matrix, respectively (Figure S12). Even though different levels of the matrix were added to the samples, the performance of the CNL model was not affected.

As for the concentration samples, TPr values of 98.7 and 99.1% were found for the 10 and 100 μg/L samples, respectively. For these samples, there was a large deviation between the FDr and reduction rates, which were 79.0 and 12.9% for the 10 μg/L sample and 59.6 and 9.2% for the 100 μg/L sample, respectively (Figure S11). From this, it seems that even though a higher reduction rate was found for lower-concentration samples, the CNL model would perform better in terms of FDr for higher-concentration samples. Moreover, since this sample set contains only two concentrations, the same evaluation was made for the matrix data set, since the matrix itself did not influence the performance and this set contained more sample concentrations. The TP, FD, and reduction rates were calculated for each concentration in this sample set, meaning the results of the no matrix, 100× dilution matrix, and 10× dilution matrix were combined for 1000, 100, 50, 25, 10, 5, 2.5, and 1 μg/L spiked internal standards. The Pearson correlation coefficient was calculated between the sample concentration and the TPr, FDr, and reduction rates, which resulted in values of 0.29, 0.34, and 0.46, respectively, showing no correlation between the sample concentration and performance. Therefore, due to the relatively low number of cases, 2149 for the 10 μg/L sample and 3702 for the 100 μg/L sample, it is more likely that the deviation is caused by the low number of cases being evaluated.

#### Fragmentation Influence

Finally, the performance of the CNL model was also evaluated for different CNL mass ranges and collision energies. For these cases, both the standard, matrix, and concentration samples are included in the assessment. For all mass ranges, with the selected CNL model parameters, a TPr between 88.8 and 91.9% was found (Figure S13). However, the FDr did increase for the higher CNL mass ranges. These FDr values were 51.1, 68.1, 74.1, and 81.1% for the CNL mass ranges 0–100, 100–200, 200–300, and 300–1000, respectively. The higher FDr performance for the larger CNL masses could be related to the relatively lower number of counts or molecules that cover that range, which can be seen in Figure S1. Additionally, the lower CNL mass range, 0–100, also has a higher overall reduction rate of 35.8%, while for the other ranges, 100–200, 200–300, and 300–1000, reduction rates of 24.8, 32.2, and 23.2%, respectively, were found. A way to account for the difference in performance for the lower and higher CNL masses could be by obtaining or expanding the number of spectra that were available for higher-mass compounds. Additionally, for higher CNL masses, there is a larger number of possible elemental combinations of which these CNLs can be comprised, leading to broader and less defined occurrence distributions for the higher CNL mass.

As for the collision energy influence, similar TP and FD rates were observed, namely, 89.4 and 66.6% for the lower collision energy and 86.6 and 67.6% for the higher collision energy, respectively (Figure S14). However, a difference in reduction rates was observed, which was 31.1.% for the low collision energy and 37.6% for the higher collision energy. When these results are compared with the trend of decreasing reduction rates for higher CNL masses, it is again confirmed. The lower collision energies, in general, create higher *m*/*z* fragments, which correspond to lower CNL masses, and, hence, perform better in eliminating TN fragments, yielding a higher reduction rate than the higher collision energy fragments.

### Comparison with Conventional Methods

An algorithm for the MS2 spectral cleanup was developed, which takes advantage of both the time domain and the mass domain. The algorithm employed CNL probability distributions and a stochastic strategy for signal cleanup. The CNL model performance was also compared with two frequently used existing techniques, namely, the apex *t*_r_ difference and correlation analysis between the precursor ion and potential fragment signals, which are dependent on the availability of time domain information. To do so, first, the optimal correlation threshold and the maximum *t*_r_ difference were determined. For the correlation, a threshold of 0.57 was found with 96.1% TPr and 60.7% FDr. Generally, correlation thresholds are set to 0.7 and higher, which could actually lead to omitting more TPs, although this is likely to depend on the quality of the signal in the data as well. If there are high fluctuations in the signal, even though a smoothing technique is used, the correlation of the true fragment peaks can be lower than the generally used threshold. As for the *t*_r_ difference, a maximum *t*_r_ difference of 0.025 was found with 96.7% TPr and 67.6% FDr. Using these parameters and the optimized 0.005 Da CNL model with a score_CNL_ threshold of 0.00, the performance of these methods and combinations were compared.

**Figure 4:**
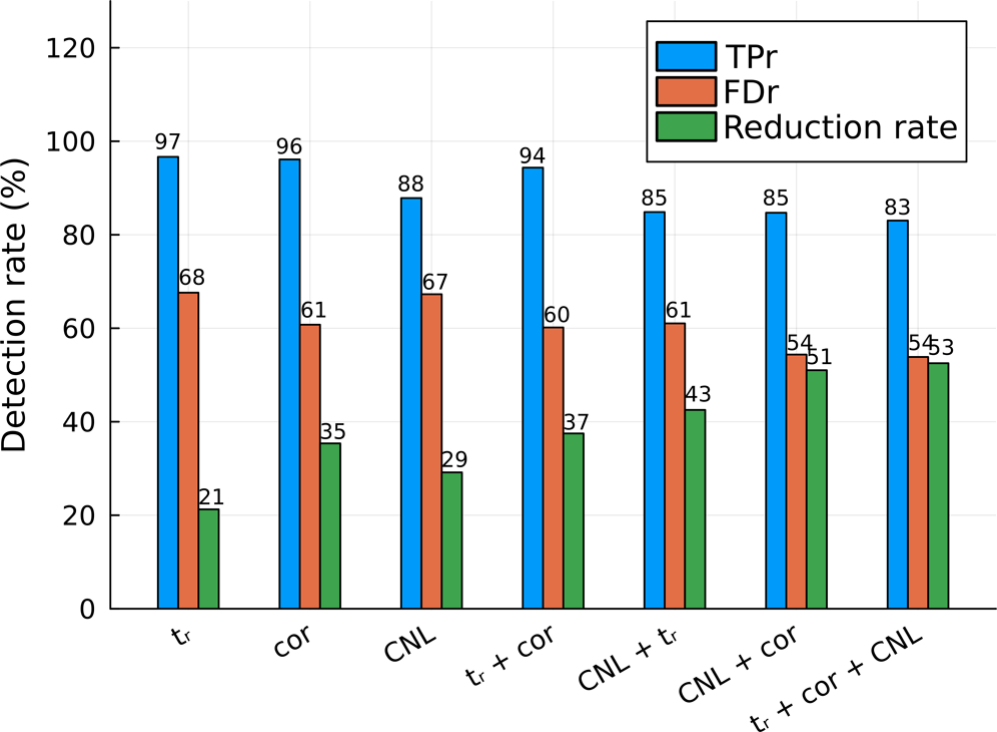
TP, FD, and reduction rates for the CNL model with a mass tolerance of 0.005 Da and a score_CNL_ of 0.00, the correlation method with a threshold of 0.57, the apex *t*_r_ method with a threshold of 0.025 min, and combinations of these three methods.

When comparing the results of using a single method, correlation analysis would be the most effective approach with a TPr of 96.1%, an FDr of 60.8%, and a reduction rate of 35.4% (Figure 4). Further reduction of the total number of features (i.e., 51.0%), while maintaining high a TPr of 84.7% and an FDr of 54.4%, can be achieved by combining the correlation analysis with the CNL model. Finally, the addition of the third method, *t*_r_ difference, does not further improve the overall performance. An increase in the reduction rate of 0.5% is mainly caused by the additional removal of 1.7% of TP cases. This can also be seen as a positive outcome, since, generally, determination of the apex *t*_r_ takes more computational power than obtaining an extracted ion chromatogram for correlation analysis. Therefore, when a time domain is available, the most effective approach for filtering fragment signals consists of a combination of correlation analysis and the CNL model proposed in this paper. However, if there is insufficient or no time domain information, the CNL model would still be able to eliminate 29.2% of the signals while retaining 87.8 % of TP fragments for these data sets.

## Conclusions

In this paper, we showed a fragment deconvolution technique that is able to clean up LC-HRMS information, using only the information of the mass domain. For the measurements used to evaluate the performance, the CNL model, with the optimized parameters of a mass tolerance of 0.005 Da and a score_CNL_ of 0.00, was able to maintain a TPr above 95%, and depending on the sample or aspect evaluated, FD rates between 33 and 79% and reduction rates between 9 and 45% have been found. Moreover, when time domain methods were combined with the CNL model, an optimal combination of correlation analysis with the CNL model was found, using a correlation threshold of 0.57 and the optimized CNL parameters. This combination was able to achieve a 51.0% reduction in the total number of fragments with a TPr of 84.7% and an FDr of 54.4%.

However, when evaluating the CNL range influence, the model performed best for the lower-range CNLs, which could be related to the higher number of TP and TN counts in this range. Therefore, it would be good if a larger number of spectra with an exact mass above 200 could be collected and included in the model. As the databases (i.e., MassBank EU, MoNA, and NIST) grow over time, the model can be easily rebuilt and optimized for the same data with the provided CNL model package. Additionally, the current model is built based on positive mode spectra. Since different fragments were found for the same chemical depending on the ionization mode, this paper focuses on the positive-mode CNL model due to the lower number of database spectra measured in negative mode. However, the developed CNLforFragments package could also be used to generate a negative-mode CNL model. Finally, when there are sufficient data available for all of the precursor ions, the potential to expand the model to obtain the likelihood of a CNL depending on the precursor ion mass could be investigated. However, there are currently too little data to implement this in the CNL model.

Overall, we showed the potential of a mass domain approach for the cleanup of fragments. The CNL model can be used when there is no time domain (e.g., for DDA) and assist existing methods when a time domain is present. Additionally, a score is calculated for each potential CNL mass, which could potentially be used as a prioritization technique to order the fragments based on true fragment likelihood. The developed algorithm is able to clean up MS2 spectra that can be fed to the structural elucidation workflows, ultimately resulting in highly confident identifications, independently from the workflow and the database. The incorporation of this model into the CompCreate.jl package for use with ULSA or other library search algorithms is an ongoing project in our group.

## Supporting Information Available

Overview of reference compounds and their corresponding sample; ROCs for the performance assessment of the CNL model using both the database and measured fragments; overview of high-probability CNLs; and case figures for TP, FN, FP, and TN detected fragments (PDF)
