# Supplementary material for: Cumulative Neutral Loss Model for Fragment Deconvolution in Electrospray Ionization High-Resolution Mass Spectrometry Data
Source: Anal Chem. 2023 Aug 7;95(33):12247–55. doi: 10.1021/acs.analchem.3c00896 (PMC10448439; doi:10.1021/acs.analchem.3c00896)
Supplement: Supplementary file 1 — ac3c00896_si_001.pdf [file ac3c00896_si_001.pdf]

Supporting Information for: Cumulative Neutral Loss Model for Fragment Deconvolution in Electrospray Ionization High-Resolution Mass Spectrometry Data

Denice van Herwerden,[*,†] Jake W. O'Brien,[‡,†] Sascha Lege,[¶] Bob W. J. Pirok,[†] Kevin V. Thomas,[‡] and Saer Samanipour[*,†,§,‡]

†*Van 't Hoff Institute for Molecular Sciences (HIMS), University of Amsterdam, Amsterdam*

‡*Queensland Alliance for Environmental Health Sciences (QAEHS), The University of Queensland, Australia*

¶*Agilent Technologies Deutschland GmbH, Waldbronn*

§*UvA Data Science Center, University of Amsterdam, Amsterdam*

E-mail: d.vanherwerden@uva.nl; s.samanipour@uva.nl

Pages: S22

Figures: 16

Tables: 2

S1 Sample composition

Table S1: List of reference compound names, formulas, exact mass, and in which sample mix it is contained.

Compound	Formula	Exact mass (Da)	Mixture
amidotrizoic acid	C11H9I3N2O4	613.770	Neochema Radiopaques Mix 5
iohexol	C19H26I3N3O9	820.880	Neochema Radiopaques Mix 5
iomeprol	C17H22I3N3O8	776.854	Neochema Radiopaques Mix 5
iopamidol	C17H22I3N3O8	776.854	Neochema Radiopaques Mix 5
iopromide	C18H24I3N3O8	790.870	Neochema Radiopaques Mix 5
clarithromycin	C38H69NO13	747.477	Neochema Radiopaques Mix 5
erythromycin	C37H67NO13	733.461	Neochema Antibiotics Mix 6
roxithromycin	C41H76N2O15	836.525	Neochema Antibiotics Mix 6
sulfamethazine	C12H14N4O2S	278.084	Neochema Antibiotics Mix 6
sulfamethoxazole	C10H11N3O3S	253.052	Neochema Antibiotics Mix 6
trimethoprim	C14H18N4O3	290.138	Neochema Antibiotics Mix 6
bezafibrate	C19H20ClNO4	361.108	Neochema Pharma Mix 17
bisoprolol	C18H31NO4	325.225	Neochema Pharma Mix 17
carbamazepine	C15H12N2O	236.095	Neochema Pharma Mix 17
clofibric acid	C10H11ClO3	214.040	Neochema Pharma Mix 17
diclofenac (free acid)	C14H11Cl2NO2	295.017	Neochema Pharma Mix 17
ibuprofen	C13H18O2	206.131	Neochema Pharma Mix 17
ketoprofen	C16H14O3	254.094	Neochema Pharma Mix 17
metoprolol	C15H25NO3	267.183	Neochema Pharma Mix 17
nadolol	C17H27NO4	309.194	Neochema Pharma Mix 17
naproxen	C14H14O3	230.094	Neochema Pharma Mix 17
phenazone (antipyrine)	C11H12N2O	188.095	Neochema Pharma Mix 17
propyphenazone	C14H18N2O	230.142	Neochema Pharma Mix 17
salbutamol	C13H21NO3	239.152	Neochema Pharma Mix 17
sotalol-HCl	C12H20N2O3S	272.119	Neochema Pharma Mix 17
atenolol	C14H22N2O3	266.163	Neochema Pharma Mix 17
propranolol	C16H21NO2	295.134	Neochema Pharma Mix 17
terbutaline	C12H19NO3	548.240	Neochema Pharma Mix 17
acephate	C4H10NO3PS	183.012	PesticideMix 1
azaconazole	C12H11Cl2N3O2	299.023	PesticideMix 1

Compound	Formula	Exact mass (Da)	Mixture
azinphos-ethyl (Guthion ethyl)	C12H16N3O3PS2	345.037	PesticideMix 1
azinphos-methyl (Guthion)	C10H12N3O3PS2	317.006	PesticideMix 1
buprofezin	C16H23N3OS	305.156	PesticideMix 1
cycloate	C11H21NOS	215.134	PesticideMix 1
cyproconazole(I)	C15H18ClN3O	291.114	PesticideMix 1
cyproconazole(II)	C15H18ClN3O	291.114	PesticideMix 1
diflufenican	C19H11F5N2O2	394.074	PesticideMix 1
dimethachlor	C13H18ClNO2	255.103	PesticideMix 1
dimoxystrobin	C19H22N2O3	326.163	PesticideMix 1
disulfoton (Ethylthiometon)*	C8H19O2PS3	274.028	PesticideMix 1
fenamiphos	C13H22NO3PS	303.106	PesticideMix 1
flufenacet (Fluthiamide) (BAY FOE 5043)	C14H13F4N3O2S	363.066	PesticideMix 1
fluopicolid	C14H8Cl3F3N2O	381.965	PesticideMix 1
fosthiazate	C9H18NO3PS2	283.047	PesticideMix 1
isofenphos methyl*	C14H22NO4PS	331.101	PesticideMix 1
isoprothiolane	C12H18O4S2	290.065	PesticideMix 1
lenacil	C13H18N2O2	234.137	PesticideMix 1
methamidophos (Metamidophos)	C2H8NO2PS	141.001	PesticideMix 1
myclobutanil	C15H17ClN4	288.114	PesticideMix 1
prochloraz	C15H16Cl3N3O2	375.031	PesticideMix 1
proquinazid	C14H17IN2O2	372.033	PesticideMix 1
spirodiclofen	C21H24Cl2O4	410.105	PesticideMix 1
spiroxamine	C18H35NO2	297.267	PesticideMix 1
tifatol (Cymiazole)*	C12H14N2S	218.088	PesticideMix 1
tralkoxydim*	C20H27NO3	329.200	PesticideMix 1
azamethiphos	C9H10ClN2O5PS	323.974	PesticideMix 2
benalaxyl	C20H23NO3	325.168	PesticideMix 2
bifenthrin*	C23H22ClF3O2	422.126	PesticideMix 2
bromuconazole(I)	C13H12BrCl2N3O	374.954	PesticideMix 2
bromuconazole(II)	C13H12BrCl2N3O	374.954	PesticideMix 2
bupirimate	C13H24N4O3S	316.157	PesticideMix 2
chlorfenvinphos(I)	C12H14Cl3O4P	357.970	PesticideMix 2
chlorfenvinphos(II)	C12H14Cl3O4P	357.970	PesticideMix 2
chlorpyrifos	C9H11Cl3NO3PS	348.926	PesticideMix 2

Compound	Formula	Exact mass (Da)	Mixture
chlorpyrifos-methyl	C7H7Cl3NO3PS	320.895	PesticideMix 2
coumaphos	C14H16ClO5PS	362.014	PesticideMix 2
diazinon (Dimpylate)	C12H21N2O3PS	304.101	PesticideMix 2
dichlorvos	C4H7Cl2O4P	219.946	PesticideMix 2
diniconazole(I)*	C15H17Cl2N3O	325.075	PesticideMix 2
diniconazole(II)	C15H17Cl2N3O	325.075	PesticideMix 2
epoxiconazole (BAS 480F)	C17H13ClFN3O	329.073	PesticideMix 2
ethion	C9H22O4P2S4	383.988	PesticideMix 2
ethoprop (Ethoprophos)	C8H19O2PS2	242.056	PesticideMix 2
fenarimol	C17H12Cl2N2O	330.033	PesticideMix 2
fenbuconazole	C19H17ClN4	336.114	PesticideMix 2
fludioxonil	C12H6F2N2O2	248.040	PesticideMix 2
fluquinconazole(I)	C16H8Cl2FN5O	375.009	PesticideMix 2
fluquinconazole(II)	C16H8Cl2FN5O	375.009	PesticideMix 2
flusilazol	C16H15F2N3Si	315.100	PesticideMix 2
hexaconazole(I)	C14H17Cl2N3O	313.075	PesticideMix 2
hexaconazole(II)	C14H17Cl2N3O	313.075	PesticideMix 2
imazalil (Enilconazole)	C14H14Cl2N2O	296.048	PesticideMix 2
ipconazole(I)	C18H24ClN3O	333.161	PesticideMix 2
ipconazole(II)	C18H24ClN3O	333.161	PesticideMix 2
metconazole(I)	C17H22ClN3O	319.145	PesticideMix 2
metconazole(II)	C17H22ClN3O	319.145	PesticideMix 2
propiconazole(I)	C15H17Cl2N3O2	341.070	PesticideMix 2
propiconazole(II)	C15H17Cl2N3O2	341.070	PesticideMix 2
tebuconazole(I) (Terbuconazole)	C16H22ClN3O	307.145	PesticideMix 2
tebuconazole(II) (Terbuconazole)	C16H22ClN3O	307.145	PesticideMix 2
tetraconazole	C13H11Cl2F4N3O	371.022	PesticideMix 2
tricyclazole	C9H7N3S	189.036	PesticideMix 2
triticonazole(I)*	C17H20ClN3O	317.129	PesticideMix 2
triticonazole(II)*	C17H20ClN3O	317.129	PesticideMix 2
uniconazole-P(I)	C15H18ClN3O	291.114	PesticideMix 2
uniconazole-P(II)	C15H18ClN3O	291.114	PesticideMix 2
vamidothion	C8H18NO4PS2	287.041	PesticideMix 2
bitertanol	C20H23N3O2	337.179	PesticideMix 3

Compound	Formula	Exact mass (Da)	Mixture
clethodim	C17H26ClNO3S	359.132	PesticideMix 3
difenconazole(I)	C19H17Cl2N3O3	405.065	PesticideMix 3
difenconazole(II)	C19H17Cl2N3O3	405.065	PesticideMix 3
etofenprox	C25H28O3	376.204	PesticideMix 3
fenhexamid	C14H17Cl2NO2	301.064	PesticideMix 3
foramsulfuron	C17H20N6O7S	452.111	PesticideMix 3
indoxacarb	C22H17ClF3N3O7	527.071	PesticideMix 3
iPC / propham	C10H13NO2	179.095	PesticideMix 3
isoxaflutole	C15H12F3NO4S	359.044	PesticideMix 3
malaoxon	C10H19O7PS	314.059	PesticideMix 3
malathion	C10H19O6PS2	330.036	PesticideMix 3
mecarbam	C10H20NO5PS2	329.052	PesticideMix 3
mepanipyrim	C14H13N3	223.111	PesticideMix 3
metalaxyl	C15H21NO4	279.147	PesticideMix 3
metazachlor	C14H16ClN3O	277.098	PesticideMix 3
methidathion	C6H11N2O4PS3	301.962	PesticideMix 3
metolachlor	C15H22ClNO2	283.134	PesticideMix 3
mevinphos (Phosdrin)*	C7H13O6P	224.045	PesticideMix 3
molinate	C9H17NOS	187.103	PesticideMix 3
oxadiazon	C15H18Cl2N2O3	344.069	PesticideMix 3
oxadixyl	C14H18N2O4	278.127	PesticideMix 3
paclobutrazol	C15H20ClN3O	293.129	PesticideMix 3
penconazole	C13H15Cl2N3	283.064	PesticideMix 3
pendimethalin (Penoxalin)	C13H19N3O4	281.138	PesticideMix 3
phenthoate (Fenthoate)*	C12H17O4PS2	320.031	PesticideMix 3
phosalone	C12H15ClNO4PS2	366.987	PesticideMix 3
phosphamidon*	C10H19ClNO5P	299.069	PesticideMix 3
picolinafen	C19H12F4N2O2	376.083	PesticideMix 3
pirimicarb	C11H18N4O2	238.143	PesticideMix 3
pirimiphos-methyl (Pirimifos-methyl)	C11H20N3O3PS	305.096	PesticideMix 3
procymidone	C13H11Cl2NO2	283.017	PesticideMix 3
profenofos	C11H15BrClO3PS	371.935	PesticideMix 3
propetamphos (Tsar)	C10H20NO4PS	281.085	PesticideMix 3
propyzamide (Pronamide)	C12H11Cl2NO	255.022	PesticideMix 3

Compound	Formula	Exact mass (Da)	Mixture
quinalphos (Diethquinalphione)	C12H15N2O3PS	298.054	PesticideMix 3
quinoxyfen	C15H8Cl2FNO	306.997	PesticideMix 3
tebufenpyrad	C18H24ClN3O	333.161	PesticideMix 3
tepraloxydim (BAS 620H)	C17H24ClNO4	341.139	PesticideMix 3
terbufos*	C9H21O2PS3	288.044	PesticideMix 3
tolclofos-methyl	C9H11Cl2O3PS	299.954	PesticideMix 3
tolylfluanide	C10H13Cl2FN2O2S2	345.978	PesticideMix 3
triadimefon	C14H16ClN3O2	293.093	PesticideMix 3
triazophos	C12H16N3O3PS	313.065	PesticideMix 3
triflumizol	C15H15ClF3N3O	345.086	PesticideMix 3
amidosulfuron	C9H15N5O7S2	369.041	PesticideMix 4
aminocarb	C11H16N2O2	208.121	PesticideMix 4
benfuracarb*	C20H30N2O5S	410.188	PesticideMix 4
boscalid (Nicobifen)	C18H12Cl2N2O	342.033	PesticideMix 4
butocarboxim*	C7H14N2O2S	190.078	PesticideMix 4
carfentrazone-ethyl	C15H14Cl2F3N3O3	411.036	PesticideMix 4
chloridazon (PAC)	C10H8ClN3O	221.036	PesticideMix 4
chlorsulfuron	C12H12ClN5O4S	357.030	PesticideMix 4
clofentezin	C14H8Cl2N4	302.013	PesticideMix 4
cyazofamid	C13H13ClN4O2S	324.045	PesticideMix 4
cymoxanil (Curzate)	C7H10N4O3	198.075	PesticideMix 4
dEET / Diethyltoluamide	C12H17NO	191.131	PesticideMix 4
diflubenzuron	C14H9ClF2N2O2	310.032	PesticideMix 4
ethirimol	C11H19N3O	209.153	PesticideMix 4
ethofumesate	C13H18O5S	286.087	PesticideMix 4
famoxadone	C22H18N2O4	374.127	PesticideMix 4
fipronil	C12H4Cl2F6N4OS	435.939	PesticideMix 4
flazasulfuron	C13H12F3N5O5S	407.051	PesticideMix 4
flufenoxuron	C21H11ClF6N2O3	488.036	PesticideMix 4
fuberidazole	C11H8N2O	184.064	PesticideMix 4
hexythiazox*	C17H21ClN2O2S	352.101	PesticideMix 4
isocarbophos*	C11H16NO4PS	289.054	PesticideMix 4
isoxaben	C18H24N2O4	332.174	PesticideMix 4
kresoxim-methyl	C18H19NO4	313.131	PesticideMix 4

Compound	Formula	Exact mass (Da)	Mixture
linuron	C9H10Cl2N2O2	248.012	PesticideMix 4
lufenuron	C17H8Cl2F8N2O3	509.978	PesticideMix 4
mandipropamid	C23H22ClNO4	411.124	PesticideMix 4
metaflumizone*	C24H16F6N4O2	506.118	PesticideMix 4
metamitron	C10H10N4O	202.085	PesticideMix 4
metrafenone	C19H21BrO5	408.057	PesticideMix 4
metribuzin	C8H14N4OS	214.089	PesticideMix 4
metsulfuron-methyl	C14H15N5O6S	381.074	PesticideMix 4
monocrotophos (Azodrin)	C7H14NO5P	223.061	PesticideMix 4
nicosulfuron	C15H18N6O6S	410.101	PesticideMix 4
novaluron	C17H9ClF8N2O4	492.012	PesticideMix 4
oxasulfuron	C17H18N4O6S	406.095	PesticideMix 4
phenmedipham	C16H16N2O4	300.111	PesticideMix 4
phoxim	C12H15N2O3PS	298.054	PesticideMix 4
prometon	C10H19N5O	225.159	PesticideMix 4
propaquizafop	C22H22ClN3O5	443.125	PesticideMix 4
propargite*	C19H26O4S	350.155	PesticideMix 4
prosulfocarb	C14H21NOS	251.134	PesticideMix 4
quinoclamine (ACN)	C10H6ClNO2	207.009	PesticideMix 4
rimsulfuron	C14H17N5O7S2	431.057	PesticideMix 4
silthiofam*	C13H21NOSSi	267.111	PesticideMix 4
teflubenzuron	C14H6Cl2F4N2O2	379.974	PesticideMix 4
thifensulfuron-methyl (DPX-M6316)	C12H13N5O6S2	387.031	PesticideMix 4
triasulfuron (Logran)	C14H16ClN5O5S	401.056	PesticideMix 4
tribenuron-methyl	C15H17N5O6S	395.090	PesticideMix 4
triflumuron	C15H10ClF3N2O3	358.033	PesticideMix 4
acetamiprid	C10H11ClN4	222.067	PesticideMix 5
alanycarb	C17H25N3O4S2	399.129	PesticideMix 5
aldicarb*	C7H14N2O2S	190.078	PesticideMix 5
azoxystrobin	C22H17N3O5	403.117	PesticideMix 5
carbendazim (Azole)	C9H9N3O2	191.069	PesticideMix 5
carboxin	C12H13NO2S	235.067	PesticideMix 5
dimethomorph(E)	C21H22ClNO4	387.124	PesticideMix 5
dimethomorph(Z)	C21H22ClNO4	387.124	PesticideMix 5

Compound	Formula	Exact mass (Da)	Mixture
diuron	C9H10Cl2N2O	232.017	PesticideMix 5
fenamidone	C17H17N3OS	311.109	PesticideMix 5
fenazaquin	C20H22N2O	306.173	PesticideMix 5
fenobucarb (Baycarb)	C12H17NO2	207.126	PesticideMix 5
fenpropidin	C19H31N	273.246	PesticideMix 5
fenpyroximate	C24H27N3O4	421.200	PesticideMix 5
imidacloprid	C9H10ClN5O2	255.052	PesticideMix 5
iprovalicarb	C18H28N2O3	320.210	PesticideMix 5
methabenzthiazuron	C10H11N3OS	221.062	PesticideMix 5
methomyl	C5H10N2O2S	162.046	PesticideMix 5
Methoxyfenozide (Intrepid)	C22H28N2O3	368.210	PesticideMix 5
oxamyl*	C7H13N3O3S	219.068	PesticideMix 5
picoxystrobin	C18H16F3NO4	367.103	PesticideMix 5
propamocarb	C9H20N2O2	188.152	PesticideMix 5
pyraclostrobin	C19H18ClN3O4	387.099	PesticideMix 5
pyridaben	C19H25ClN2OS	364.138	PesticideMix 5
pyridate	C19H23ClN2O2S	378.117	PesticideMix 5
pyriproxyfen (Pyriproxifen)	C20H19NO3	321.136	PesticideMix 5
tebufenozide	C22H28N2O2	352.215	PesticideMix 5
thiabendazole	C10H7N3S	201.036	PesticideMix 5
thiacloprid	C10H9ClN4S	252.024	PesticideMix 5
thiamethoxam	C8H10ClN5O3S	291.019	PesticideMix 5
thiodicarb	C10H18N4O4S3	354.049	PesticideMix 5
thiofanox*	C9H18N2O2S	218.109	PesticideMix 5
trifloxystrobin	C20H19F3N2O4	408.130	PesticideMix 5
2,3,5-trimethacarb (Landrin)	C11H15NO2	193.110	PesticideMix 6
carbaryl	C12H11NO2	201.079	PesticideMix 6
carbosulfan*	C20H32N2O3S	380.213	PesticideMix 6
diethofencarb	C14H21NO4	267.147	PesticideMix 6
fenoxycarb	C17H19NO4	301.131	PesticideMix 6
flonicamid	C9H6F3N3O	229.046	PesticideMix 6
flumioxazin	C19H15FN2O4	354.102	PesticideMix 6
furathiocarb*	C18H26N2O5S	382.156	PesticideMix 6
mesosulfuron-methyl	C17H21N5O9S2	503.078	PesticideMix 6

Compound	Formula	Exact mass (Da)	Mixture
omethoate	C5H12NO4PS	213.022	PesticideMix 6
pencycuron	C19H21ClN2O	328.134	PesticideMix 6
phosmet (Imidan)	C11H12NO4PS2	316.995	PesticideMix 6
propoxur	C11H15NO3	209.105	PesticideMix 6
pyrimethanil	C12H13N3	199.111	PesticideMix 6
spiromesifen*	C23H30O4	370.214	PesticideMix 6
spirotetramat	C21H27NO5	373.189	PesticideMix 6
sulfentrazone	C11H10Cl2F2N4O3S	385.982	PesticideMix 6
triadimenol	C14H18ClN3O2	295.109	PesticideMix 6
trichlorfon (Dylox) (DEP)	C4H8Cl3O4P	255.923	PesticideMix 6
trietazine	C9H16ClN5	229.109	PesticideMix 6
zoxamide	C14H16Cl3NO2	335.025	PesticideMix 6
avermectin B1a (Abamectin B1a)*	C48H72O14	872.492	PesticideMix 7
avermectin B1b (Abamectin B1b)*	C47H70O14	858.477	PesticideMix 7
benzoximate*	C18H18ClNO5	363.087	PesticideMix 7
bispyribac	C19H18N4O8	430.112	PesticideMix 7
chloroxuron	C15H15ClN2O2	290.082	PesticideMix 7
chlortoluron (Chlorotoluron)	C10H13ClN2O	212.072	PesticideMix 7
cycluron	C11H22N2O	198.173	PesticideMix 7
dinotefuran	C7H14N4O3	202.107	PesticideMix 7
dioxacarb	C11H13NO4	223.084	PesticideMix 7
fenuron (N,N-Dimethyl-N-phenylurea)	C9H12N2O	164.095	PesticideMix 7
flubendiamide	C23H22F7IN2O4S	682.023	PesticideMix 7
forchlorfenuron	C12H10ClN3O	247.051	PesticideMix 7
furalaxyl	C17H19NO4	301.131	PesticideMix 7
hexaflumuron	C16H8Cl2F6N2O3	459.982	PesticideMix 7
hydramethylnon	C25H24F6N4	494.191	PesticideMix 7
ivermectin B1a*	C48H74O14	874.508	PesticideMix 7
ivermectin B1b*	C47H72O14	860.492	PesticideMix 7
methiocarb (Mercaptodimethur)	C11H15NO2S	225.082	PesticideMix 7
methoprotryne	C11H21N5OS	271.147	PesticideMix 7
mexacarbate (Zectran)	C12H18N2O2	222.137	PesticideMix 7
moxidectin (Cydectin)	C37H53NO8	639.377	PesticideMix 7
nitenpyram	C11H15ClN4O2	270.088	PesticideMix 7

Compound	Formula	Exact mass (Da)	Mixture
promecarb	C12H17NO2	207.126	PesticideMix 7
pymetrozine	C10H11N5O	217.096	PesticideMix 7
pyracarbolid	C13H15NO2	217.110	PesticideMix 7
quinmerac	C11H8ClNO2	221.024	PesticideMix 7
rotenone	C23H22O6	394.142	PesticideMix 7
secbumeton	C10H19N5O	225.159	PesticideMix 7
spinosyn A	C41H65NO10	731.461	PesticideMix 7
spinosyn D*	C42H67NO10	745.477	PesticideMix 7
tebuthiuron	C9H16N4OS	228.104	PesticideMix 7
temephos (Abate)	C16H20O6P2S3	465.990	PesticideMix 7
Thidiazuron	C9H8N4OS	220.042	PesticideMix 7
beflubutamid	C18H17F4NO2	355.120	PesticideMix 8
bifenazate (D 2341)	C17H20N2O3	300.147	PesticideMix 8
carbofuran	C12H15NO3	221.105	PesticideMix 8
chlorantraniliprole*	C18H14BrCl2N5O2	480.971	PesticideMix 8
clomazone	C12H14ClNO2	239.071	PesticideMix 8
cyprodinil	C14H15N3	225.127	PesticideMix 8
desmedipham	C16H16N2O4	300.111	PesticideMix 8
dimethoate	C5H12NO3PS2	229.000	PesticideMix 8
ethidimuron (Sulfadiazole)	C7H12N4O3S2	264.03508	PesticideMix 8
ethoxyquin	C14H19NO	217.147	PesticideMix 8
fluazinam (Shirlan)	C13H4Cl2F6N4O4	463.951	PesticideMix 8
flumetsulam	C12H9F2N5O2S	325.045	PesticideMix 8
fluometuron	C10H11F3N2O	232.082	PesticideMix 8
fluoxastrobin	C21H16ClFN4O5	458.079	PesticideMix 8
flutriafol	C16H13F2N3O	301.103	PesticideMix 8
halofenozide	C18H19ClN2O2	330.114	PesticideMix 8
halosulfuron-methyl	C13H15ClN6O7S	434.04115	PesticideMix 8
methacrifos*	C7H13O5PS	240.02213	PesticideMix 8
Metobromuron	C9H11BrN2O2	258.000	PesticideMix 8

* Compounds that were not included in the suspect list due absence of reference spectra in the databases.

S2 CNL Model

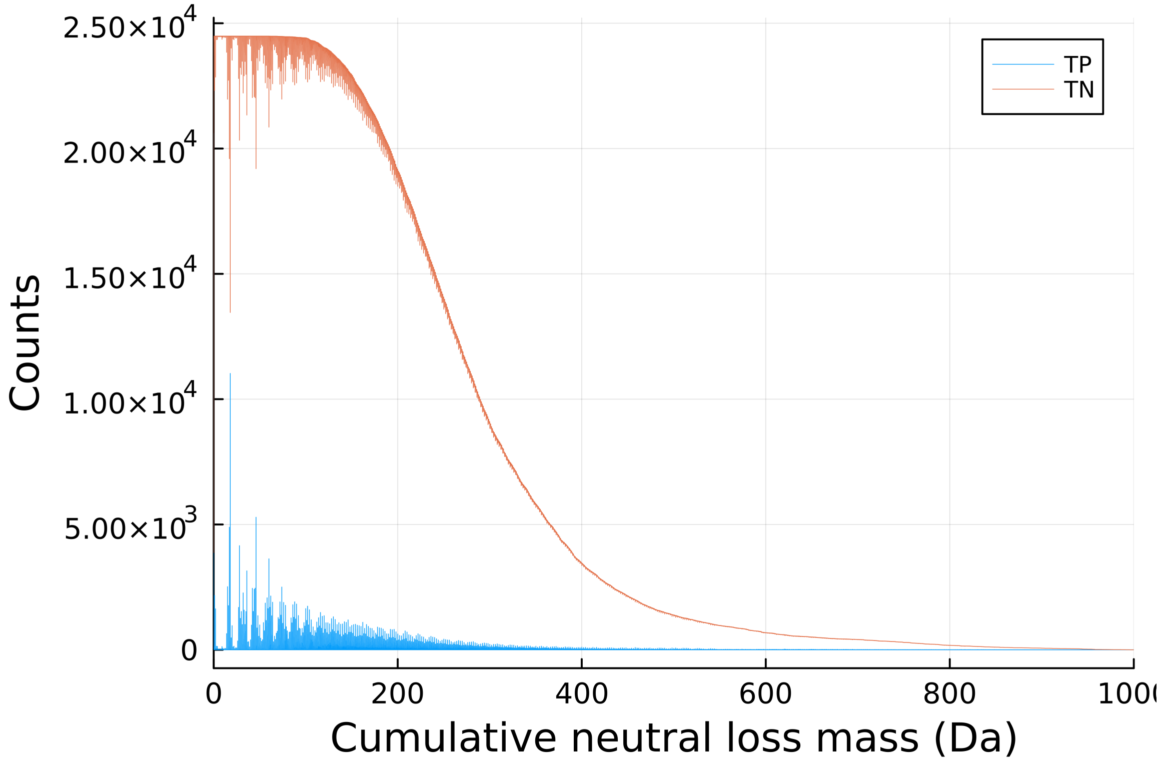

Figure S1: The total number of TP and TN counts for each CNL bin (i.e., 0.001 Da).

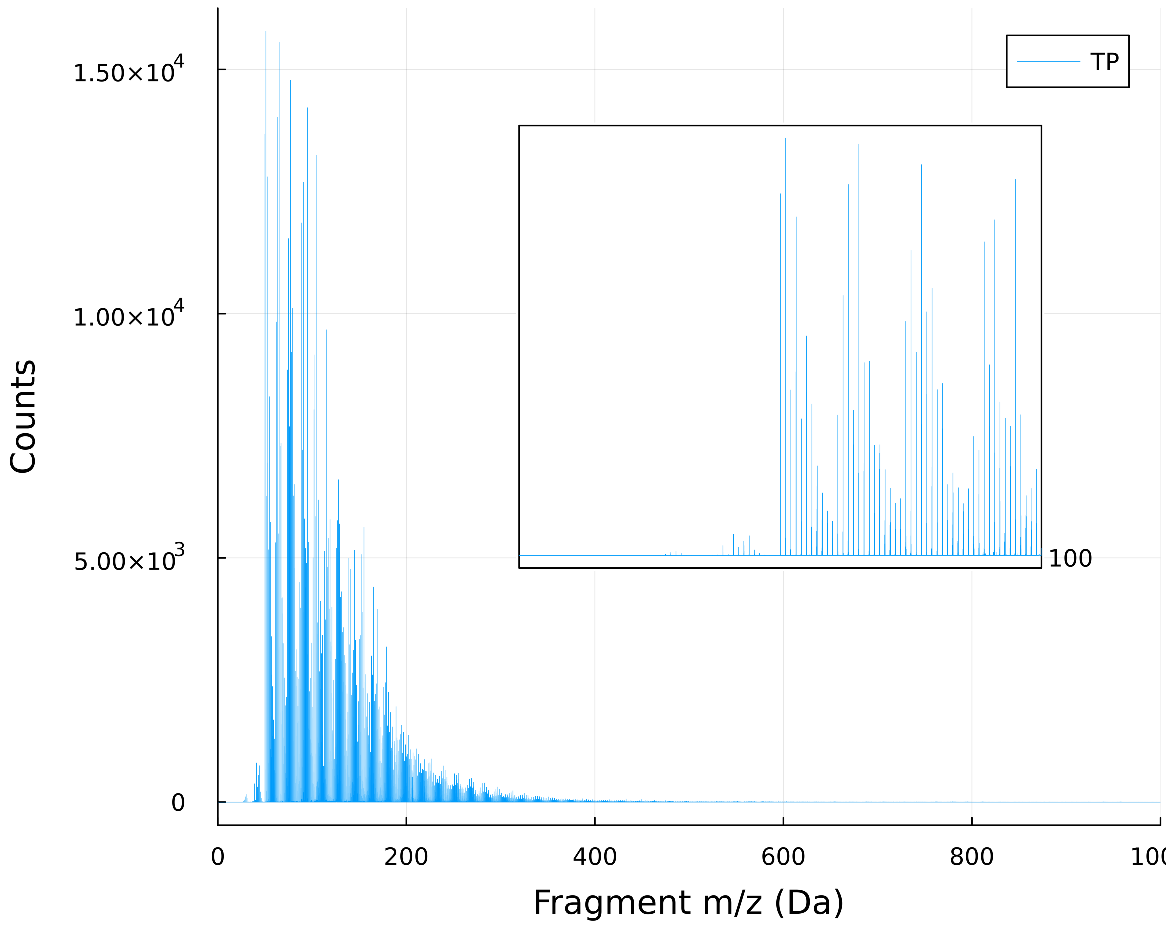

Figure S2: The total number of TP fragment m/z counts for each bin from 0 to 1000 m/z with a step size of 0.001 Da. A) shows the full distribution and B) shows the distribution from 0 to 100 Da.

Table S2: Overview of CNLs with high TP probabilities and their corresponding potential CHNO-based elemental composition.

$P(TP)$	$P(TN)$	CNL mass	Formula	Exact mass
0.005046	3.17E-05	18.0125	H2O	18.0106
0.002260	3.16E-05	46.0075	CH2O2	46.0054
0.002241	3.51E-05	17.0295	NH3	17.0266
0.001684	3.58E-05	27.9975	N2	28.0062
0.001607	3.24E-05	60.0225	C2H4O2	60.0211
0.001207	3.29E-05	15.0255	CH3	15.0235
0.001186	3.64E-05	28.0005	N2	28.0062
0.001126	3.30E-05	42.0115	C2H2O	42.0106
0.001071	3.65E-05	74.0395	CH4N3O	74.0355
			C3H6O2	74.0368
0.001008	3.32E-05	32.0275	CH4O	32.0262
0.001008	3.32E-05	44.0275	C2H4O	44.0262

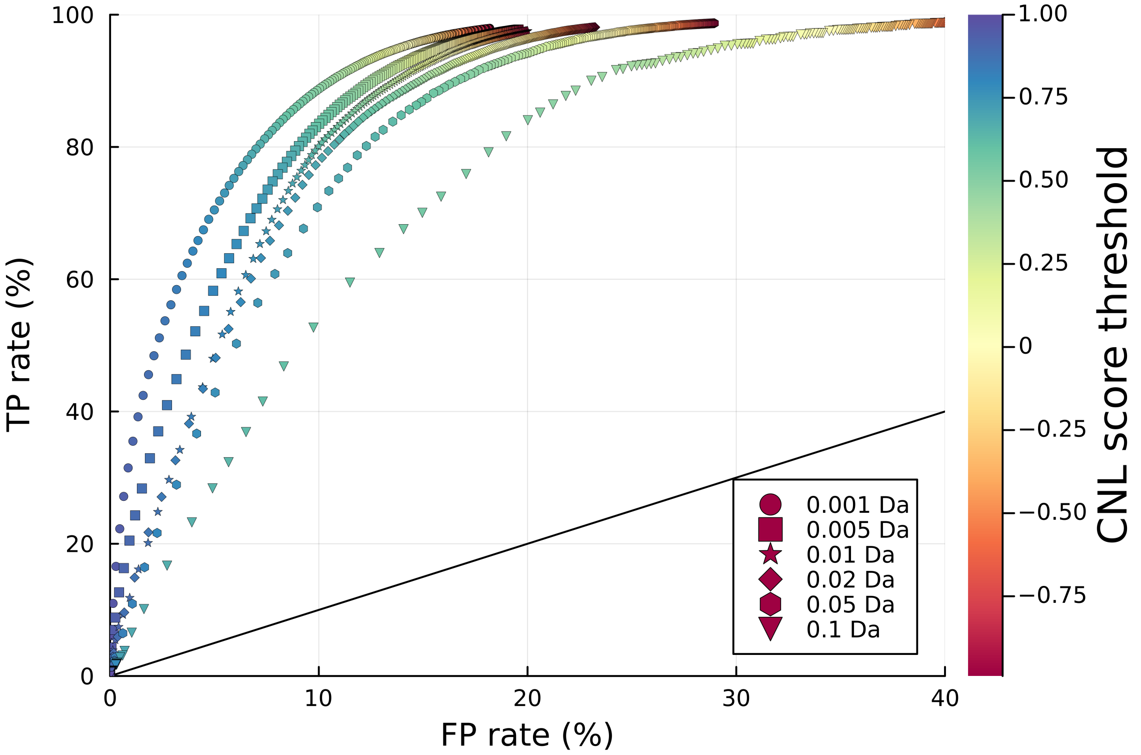

Figure S3: Receiver operator curve for the TP and FP rates of the CNL model for the database fragments, using different mass tolerances and score$_{CNL}$ thresholds represented by different shapes and colors, respectively. Additionally, the black line represents the 1:1 ratio between TPr and FPr.

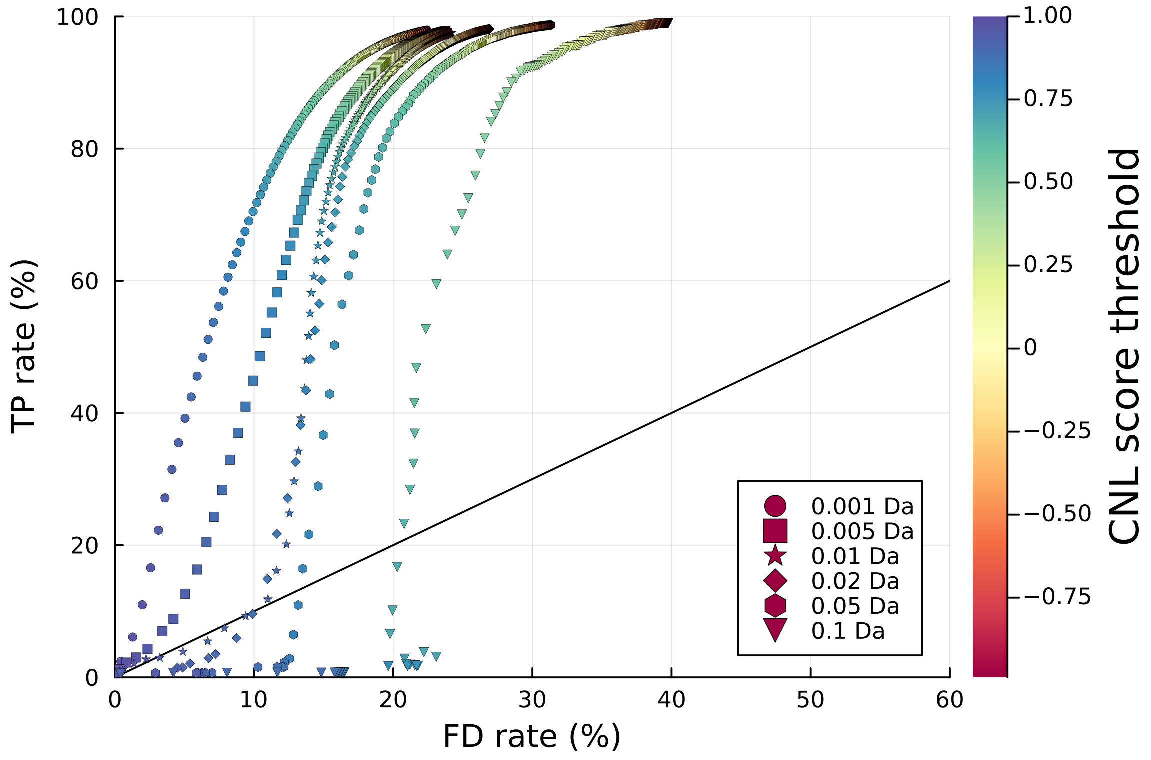

Figure S4: Receiver operator curve for the *TP* and *FD* rates of the CNL model for the database fragments, using different mass tolerances and $score_{CNL}$ thresholds represented by different shapes and colors, respectively. Additionally, the black line represents the 1:1 ratio between *TPr* and *FDr*.

S3 Real samples

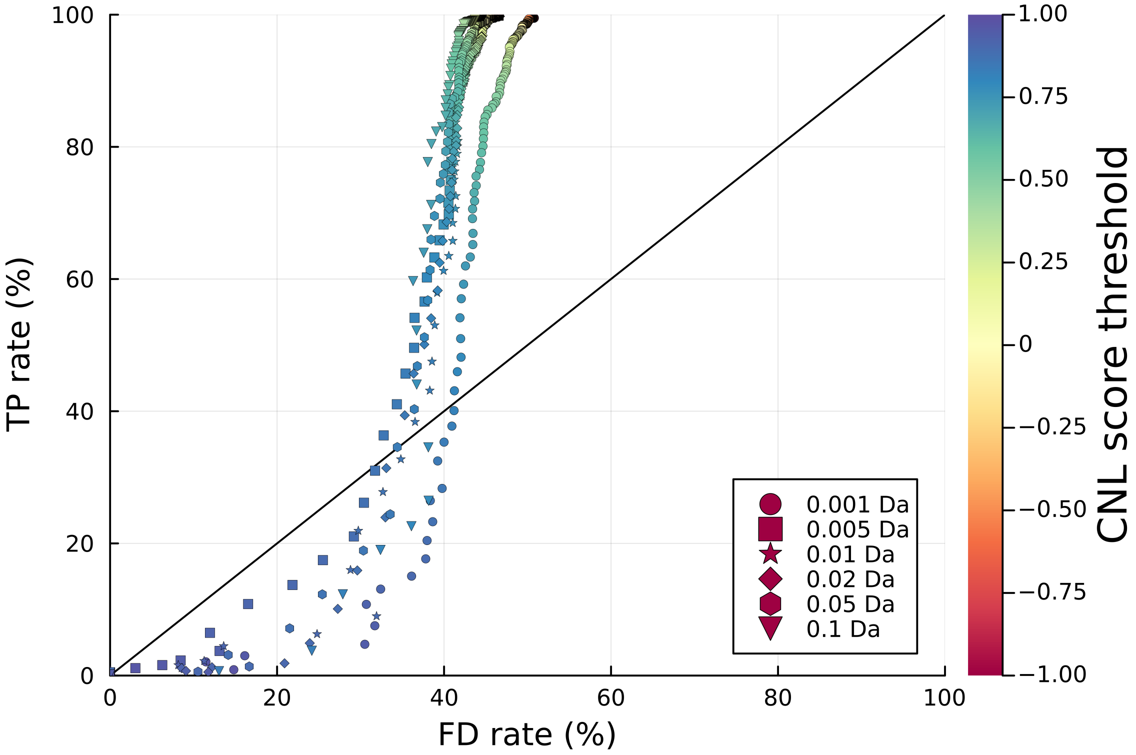

Figure S5: Receiver operator curve for the TP and FD rates of the CNL model for the real samples, using different mass tolerances and $score_{CNL}$ thresholds represented by different shapes and colors, respectively.

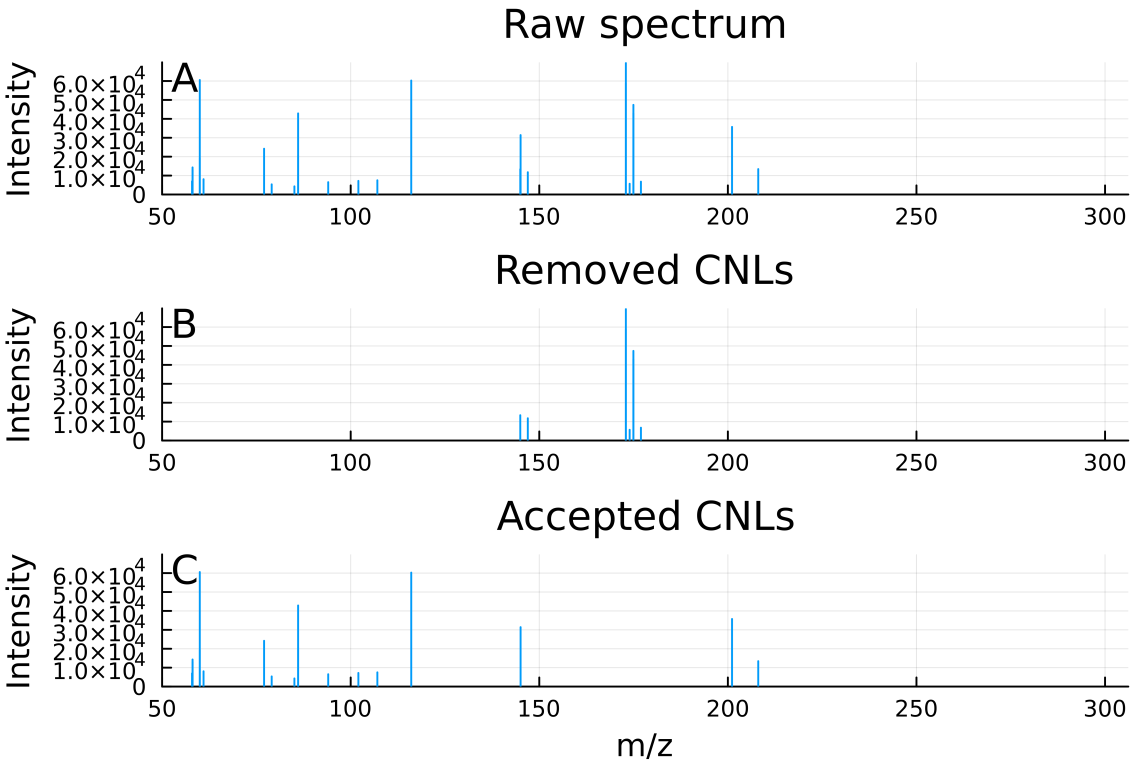

Figure S6: A) shows all detected signals within a single measurement for buprofezin, B) shows the signals that were removed according to the CNL model with a mass tolerance of 0.005 and a score threshold of 0.00, and C) shows the cleaned spectrum.

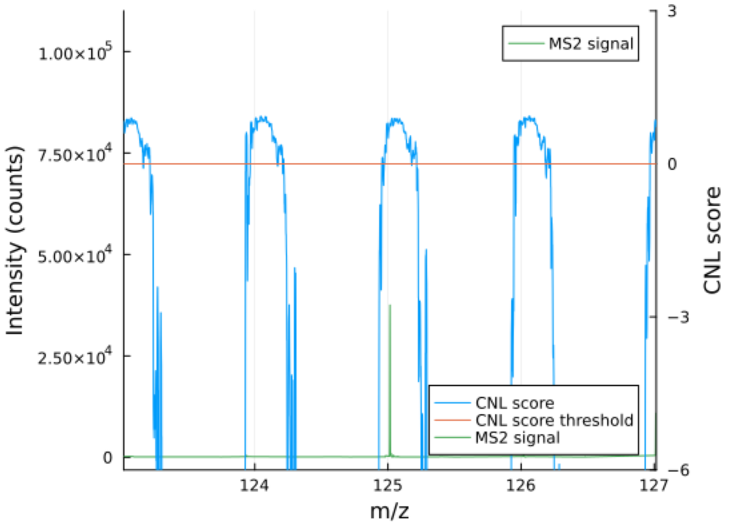

Figure S7: Extracted signal for a TP detected fragment with a m/z of 125.016 Da (CNL of 167.109) for Cyproconazole(I) plotted in green with the intensity on the left y-axis. Additionally, the score$_{CNL}$ and threshold were plotted on the right y-axis in blue and orange, respectively.

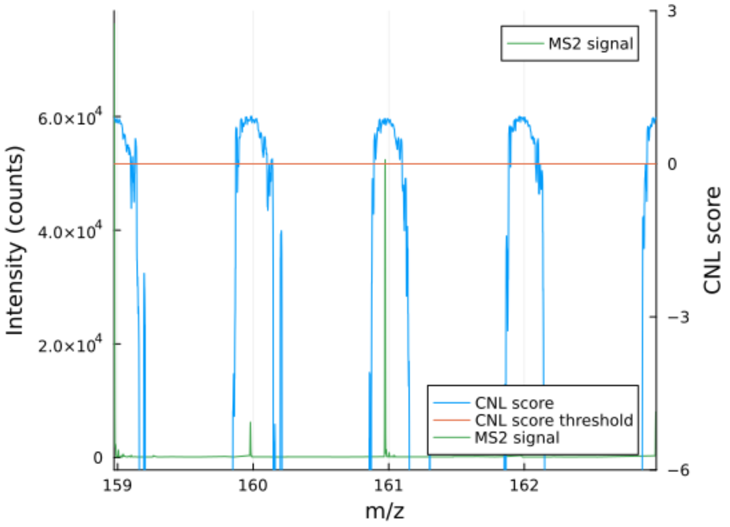

Figure S8: Extracted signal for a FP detected fragment with a m/z of 160.974 Da (CNL of 139.059) for Azaconazole plotted in green with the intensity on the left y-axis. Additionally, the score$_{CNL}$ and threshold were plotted on the right y-axis in blue and orange, respectively.

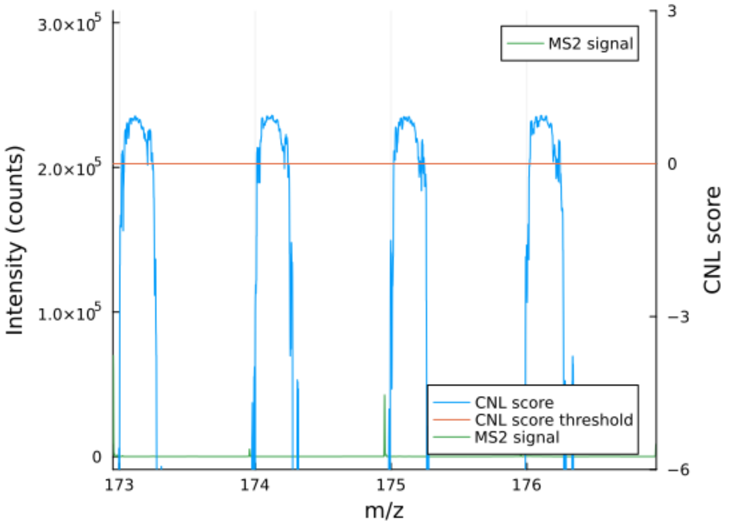

Figure S9: Extracted signal for a TN detected fragment with a m/z of 174.952 Da (CNL of 131.215) for Buprofezin plotted in green with the intensity on the left y-axis. Additionally, the score$_{CNL}$ and threshold were plotted on the right y-axis in blue and orange, respectively.

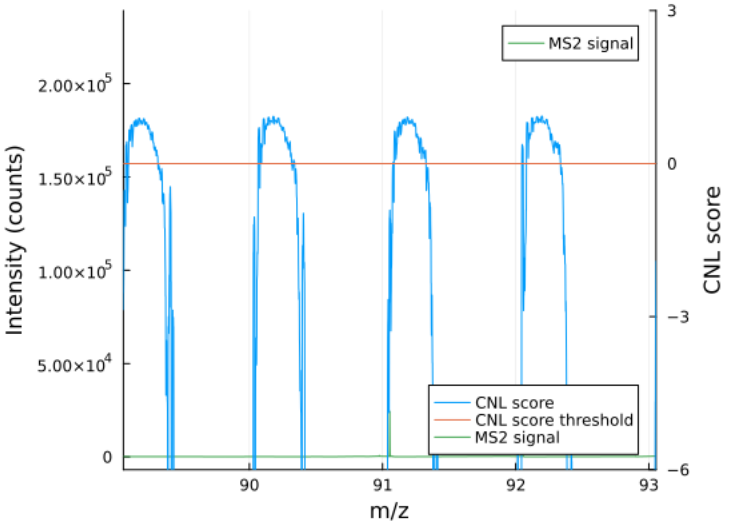

Figure S10: Extracted signal for a FN detected fragment with a m/z of 91.054 Da (CNL of 183.200) for Fenpropidin plotted in green with the intensity on the left y-axis. Additionally, the $score_{CNL}$ and threshold were plotted on the right y-axis in blue and orange, respectively.

S3.1 Concentration Influence

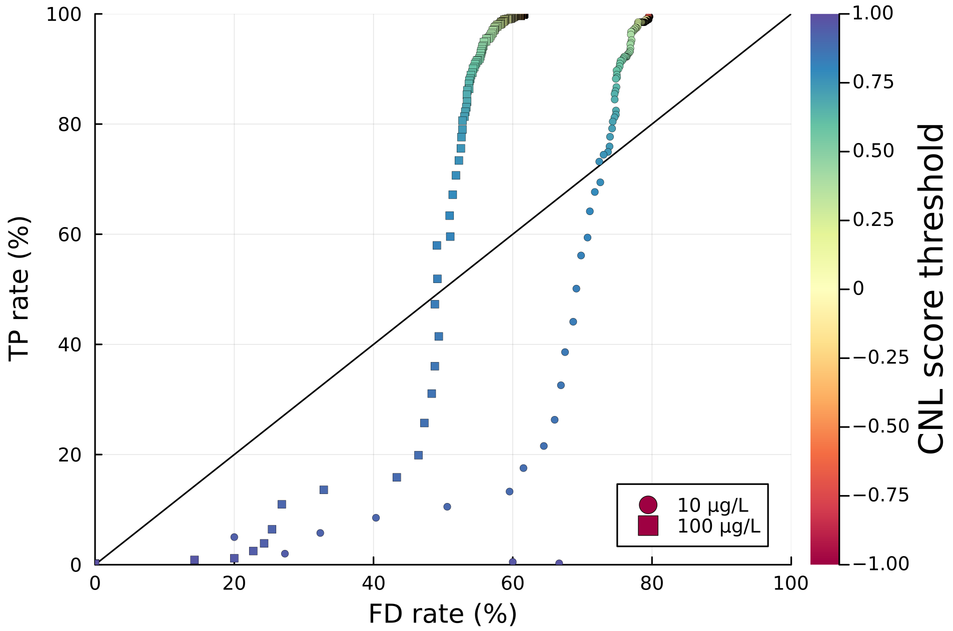

Figure S11: Receiver operator curve for the *TP* and *FD* rates of the CNL model for real samples with a varying $score_{CNL}$ threshold. Here the difference for results with low spiked concentration (circles) and high concentration (squares) can be seen. Additionally, the black line represents the 1:1 ratio between *TPr* and *FDr*.

S3.2 Matrix Influence

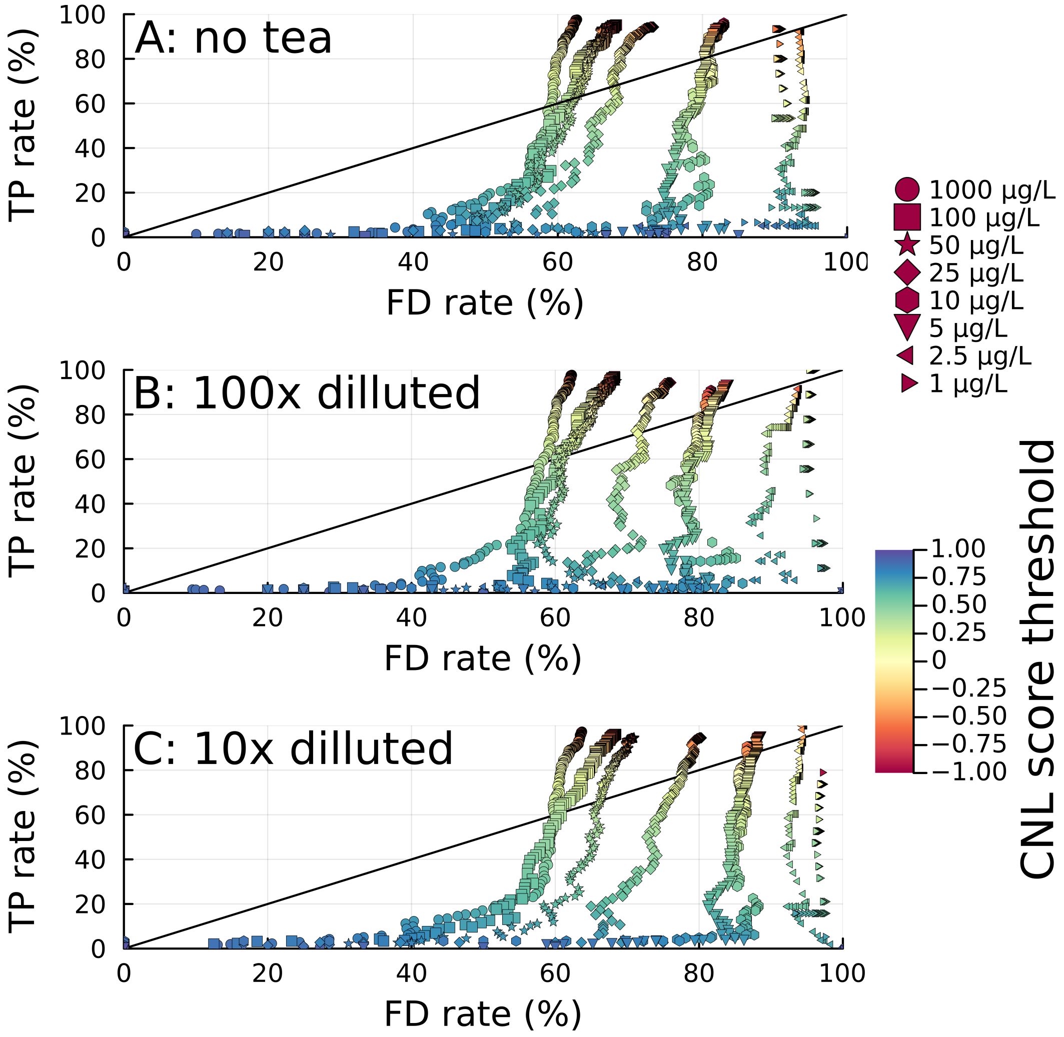

Figure S12: Receiver operator curves for the *TP* and *FD* rates of the CNL model for real samples with a varying $score_{CNL}$ threshold. Here the difference for results with no added matrix (**A**), 100 times diluted tea (**B**), and 10 times diluted tea (**C**) can be seen. Each subplot contains multiple ROC curves, each for a different concentration of added standards. Additionally, the black line represents the 1:1 ratio between *TPr* and *FDr*.

S3.3 CNL Range Influence

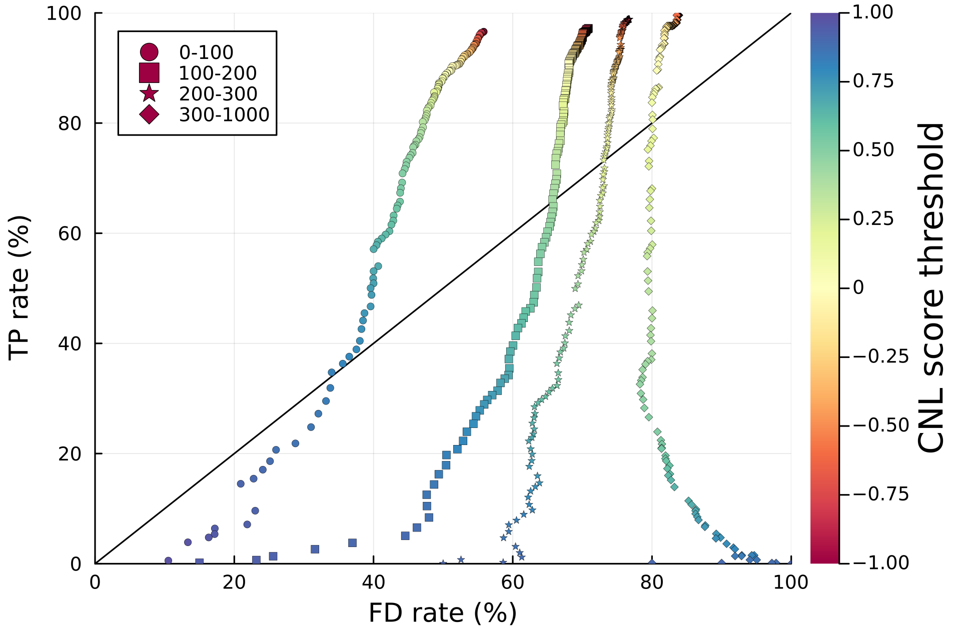

Figure S13: Receiver operator curve for the TP and FD rates of the CNL model for real samples with a varying score_{CNL} threshold. Here the performance for 4 CNL ranges can be seen. Additionally, the black line represents the 1:1 ratio between TPr and FDr.

S3.4 Collision Energy Influence

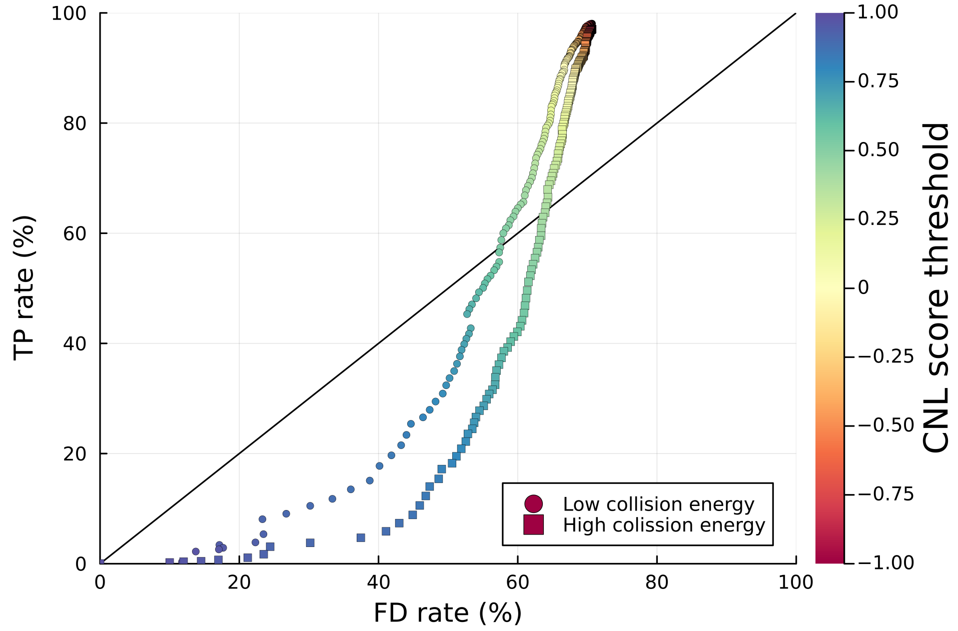

Figure S14: Receiver operator curve for the TP and FD rates of the CNL model for real samples with a varying score$_{CNL}$ threshold. Here the difference for results with low collision energy (circles) and high collision energy (squares) can be seen. Additionally, the black line represents the 1:1 ratio between TPr and FDr.

S4 Comparison with Conventional Method

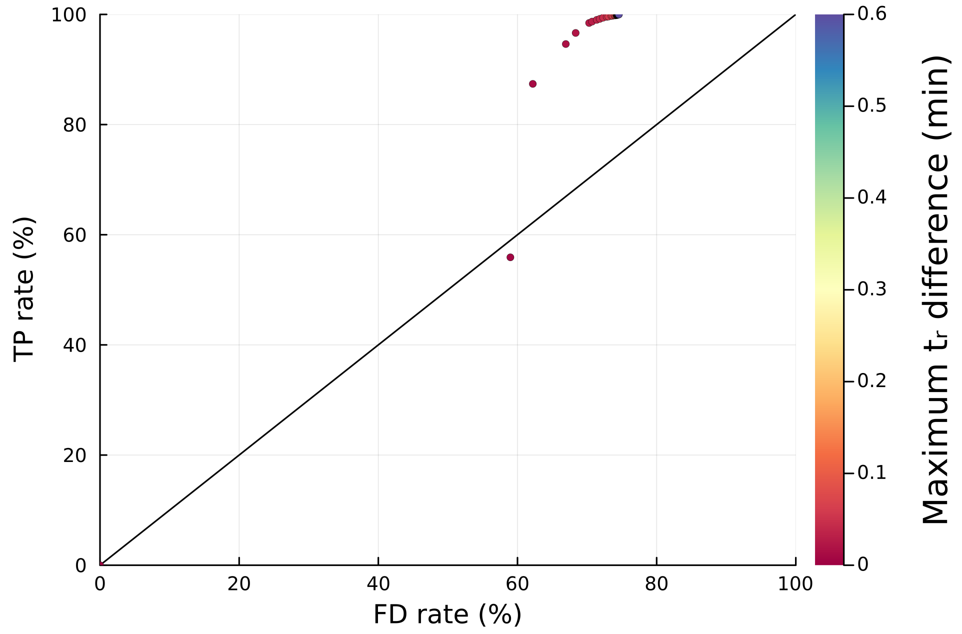

Figure S15: Receiver operator curve for the *TP* and *FD* rates of the apex retention time difference method for real samples with a varying maximum retention time difference. Additionally, the black line represents the 1:1 ratio between *TPr* and *FDr*.

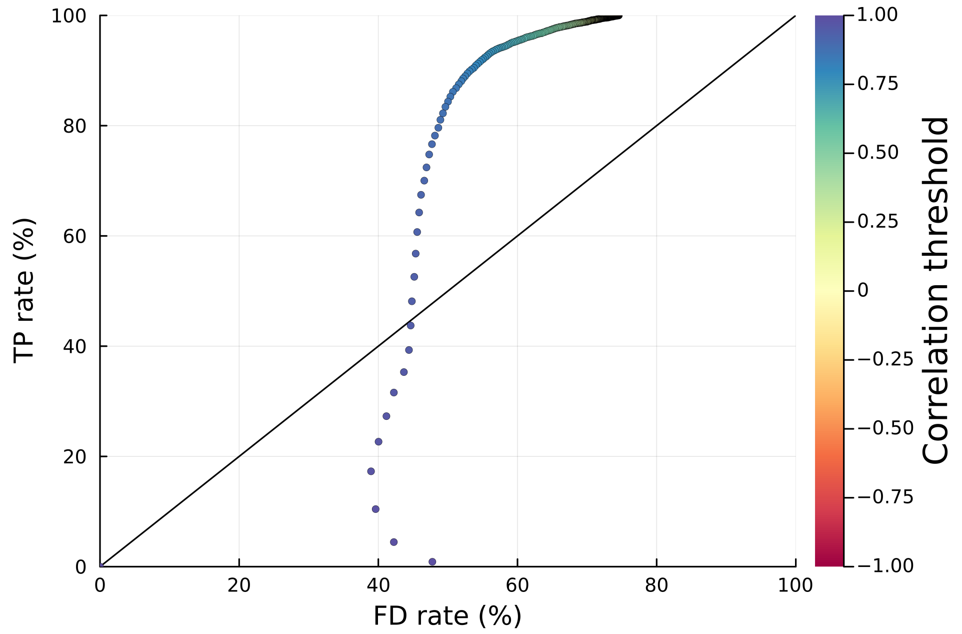

Figure S16: Receiver operator curve for the *TP* and *FD* rates of the correlation method for real samples with a varying minimum correlation. Additionally, the black line represents the 1:1 ratio between *TPr* and *FDr*.
